# Survival and Clinicopathological Significance of SIRT1 Expression in Cancers: A Meta-Analysis

**DOI:** 10.3389/fendo.2019.00121

**Published:** 2019-03-13

**Authors:** Min Sun, Mengyu Du, Wenhua Zhang, Sisi Xiong, Xingrui Gong, Peijie Lei, Jin Zha, Hongrui Zhu, Heng Li, Dong Huang, Xinsheng Gu

**Affiliations:** ^1^Department of General Surgery, Taihe Hospital, Hubei University of Medicine, Shiyan, China; ^2^Department of Anesthesiology, Institute of Anesthesiology, Taihe Hospital, Hubei University of Medicine, Shiyan, China; ^3^Department of Pharmaceutical Sciences, College of Pharmacy, Hubei University of Medicine, Shiyan, China; ^4^School of Nursing, Hubei University of Medicine, Shiyan, China; ^5^The First Clinical School, Hubei University of Medicine, Shiyan, China; ^6^Department of Pharmacology, College of Basic Medical Sciences, Hubei University of Medicine, Shiyan, China

**Keywords:** SIRT1, cancer, prognosis, clinicopathological significance, meta-analysis

## Abstract

**Background:** Silent information regulator 2 homolog 1 (SIRT1) is an evolutionarily conserved enzymes with nicotinamide adenine dinucleotide (NAD)^+^-dependent deacetylase activity. SIRT1 is involved in a large variety of cellular processes, such as genomic stability, energy metabolism, senescence, gene transcription, and oxidative stress. SIRT1 has long been recognized as both a tumor promoter and tumor suppressor. Its prognostic role in cancers remains controversial.

**Methods:** A meta-analysis of 13,138 subjects in 63 articles from PubMed, EMBASE, and Cochrane Library was performed to evaluate survival and clinicopathological significance of SIRT1 expression in various cancers.

**Results:** The pooled results of meta-analysis showed that elevated expression of SIRT1 implies a poor overall survival (OS) of cancer patients [Hazard Ratio (HR) = 1.566, 95% CI: 1.293–1.895, *P* < 0.0001], disease free survival (DFS) (HR = 1.631, 95% CI: 1.250–2.130, *P* = 0.0003), event free survival (EFS) (HR = 2.534, 95% CI: 1.602–4.009, *P* = 0.0001), and progress-free survival (PFS) (HR = 3.325 95% CI: 2.762–4.003, *P* < 0.0001). Elevated SIRT1 level was associated with tumor stage [Relative Risk (RR) = 1.299, 95% CI: 1.114–1.514, *P* = 0.0008], lymph node metastasis (RR = 1.172, 95% CI: 1.010–1.360, *P* = 0.0363), and distant metastasis (RR = 1.562, 95% CI: 1.022–2.387, *P* = 0.0392). Meta-regression and subgroup analysis revealed that ethnic background has influence on the role of SIRT1 expression in predicting survival and clinicopathological characteristics of cancers. Overexpression of SIRT1 predicted a worse OS and higher TNM stage and lymphatic metastasis in Asian population especially in China.

**Conclusion:** Our data suggested that elevated expression of SIRT1 predicted a poor OS, DFS, EFS, PFS, but not for recurrence-free survival (RFS) and cancer-specific survival (CCS). SIRT1 overexpression was associated with higher tumor stage, lymph node metastasis, and distant metastasis. SIRT1-mediated molecular events and biological processes could be an underlying mechanism for metastasis and SIRT1 is a therapeutic target for inhibiting metastasis, leading to good prognosis.

## Introduction

Silent information regulator 2 homolog 1 (SIRT1) is an evolutionarily conserved enzymes with nicotinamide adenine dinucleotide (NAD)^+^-dependent deacetylase activity and a member of the mammalian sirtuin family. It is expressed in almost all human tissues and localized in both nuclei and cytoplasm (1). Its substrates include histones and non-histone proteins such as transcription factors (2–4). SIRT1 is involved in a large variety of cellular processes, such as genomic stability, energy metabolism, senescence, gene transcription, and oxidative stress (5). It has been shown to be involved in a spectrum of diseases, including cancer, diabetes, obesity, and neurodegenerative diseases (6–8). SIRT1 plays an important role in regulating glucose and lipid metabolism and regulates malignancy in tumors (9).

SIRT1 has long been recognized as both a tumor promoter and tumor suppressor (10–12). This is also shown in recent studies. SIRT1 promotes proliferation, migration, and invasion of breast cancer cell line MCF-7 (13). SIRT1 promotes proliferation and paclitaxel-resistance of human cervical cancer cells (14). Yang et al. found that SIRT1 levels are lower in non-small cell lung cancer (NSCLC) than the normal control group (15), but Gharabaghi et al. found that SIRT1 are over expressed in NSCLC (16). The role of SIRT1 in prognosis of cancer was also investigated in several studies. Over expression of SIRT1 suggests poor prognosis in luminal breast cancer (17) and serous epithelial ovarian cancer (EOC) (18), gastric cancer (19), high pathological stage and worse overall survival in the lung adenocarcinoma patients (20), decreased survival and increased relapse in breast cancer patients (3, 21), colorectal carcinoma patients (22), lymphangiogenesis, lymphovascular invasion, and prognosis in pN0 esophageal squamous cell carcinoma (23), soft tissue sarcomas (24), both operable triple-negative and non-triple-negative breast cancer (25), hepatocellular carcinoma (26), gastric carcinoma (27), diffuse large B-cell lymphoma (28). On the other hand, SIRT1 expression is found to be associated with good prognosis for head and neck squamous cell carcinoma patients (29), and colorectal cancer (30). Therefore, the prognostic and clinicopathological significance of SIRT1 abnormal expression in cancers remain to be elucidated.

Prognostic value and clinicopathological association of SIRT1 with cancers have been analyzed in previous meta-analysis (31–36). However, the studies included in these meta-analysis were limited to mostly Asian population, single or several cancer types, or they were published several years ago (31–36). In the present study, we conducted an updated and more comprehensive meta-analysis and subgroup analysis to reveal the prognostic value and clinicopathological association of SIRT1 abnormal expression in cancers.

## Methods

### Search Strategy

We retrieved literature published in between 1966 and April 1st, 2018 by searching PubMed, EMBASE, and Cochrane Library with the keywords (1) “SIRT1” OR “sirtuin 1” OR “SIR2” OR “SIR2L1” OR “SIR2alpha” OR “silent mating type information regulation 2 homolog-1” AND (2) “tumor OR cancer OR carcinoma OR neoplasm” and using the search strategies as illustrated in Supplementary Tables 1A–C. We selected and evaluated all relevant studies and review articles about SIRT1 and inquired the authors for unpublished raw data. The search and selection of articles for the study were separately conducted based on a common set of criteria. The divergence in opinion were settled through discussion among ourselves.

### Inclusion and Exclusion Criteria

This meta-analysis was conducted according to Meta-analysis of Observational Studies in Epidemiology (MOOSE) Checklist. Studies enrolled in this analysis satisfied the following requirements: (i) patients must be diagnosed with cancer via pathology; (ii) The expression of SIRT1 must be determined by quantitative real-time polymerase chain reaction (q-PCR), immunohistochemistry (IHC), or *in situ* hybridization (ISH); (iii) The correlation between SIRT1 expression and prognosis or clinicopathological features was investigated; (iv) The Hazard Ratio (HR) and its 95% confidence interval (CI) for survival indicator on the basis of SIRT1 expression level were readily available or could be calculated indirectly; (v) The most representative and most accurate study was adopted when a single sample source was used in multiple studies to avoid unnecessary cohort overlapping. Studies that have satisfied the abovementioned inclusion requirements were further ruled out if they had any of the following flaws: (i) duplicated articles or data; (ii) not human studies; (iii) review articles or letters; (iv) lack of sufficient data or information to get HR; (v) articles not written in English.

### Quality Assessment of Included Studies

We used the Newcastle-Ottawa Scale (NOS) to assess the quality of each included study. Scores ≥ 7 were considered high quality. We used a “star system” for case-control studies (Supplementary Table 2).

### Data Extraction

We extracted the following data from the full texts of eligible studies: (i) the first author; (ii) publication year; (iii) characteristics of the studies, which comprised of the patients' nationality, sample size, tumor type, and clinicopathological characteristics; (iv) the assay method and cut-off value of SIRT1; (v) HRs of SIRT1 expression for OS, disease-free survival (DFS), event-free survival (EFS), recurrence-free survival (RFS), cancer-specific survival (CCS), progression-free survival (PFS); (vi) if the HR for OS, DFS, EFS, RFS, CCS and PFS were calculated by both univariate and multivariate analyses, the latter was our first choice, given that these results were adjusted for confounding factors. If a study did not report the HR, we estimated HR and their corresponding 95% CI using the method described by Parmar et al. (37) and Tierney et al. (38). We recovered the data of Kaplan-Meier curves via the Engauge Digitizer version 9.8 (http://markummitchell.github.io/engauge-digitizer) and calculated the HR and its 95% CI. We repeated this process three times to reduce variability. Any divergence regarding the extraction and interpretation of all data was resolved by discussion among ourselves until consensus was reached.

### Statistical Analysis

All the HRs and their 95% CIs were combined to evaluate the effect of SIRT1 high expression on prognosis. If the pooled HR < 1 and their 95% CI did not overlap the invalid line in the forest plot, the high expression of SIRT1 predicted a good OS. If the 95% CI overlapped the invalid line, the combined HR was considered insignificant. Otherwise, the combined HR predicted a poor OS. The heterogeneity of the pooled results was examined via Cochrane's Q test and Higgins' I-squared, and *P* < 0.1 or I^2^ ≥ 25% was considered high heterogeneity. If *P* > 0.1 and I^2^ < 25%, we ignored the influence of heterogeneity and pooled the overall result using a fixed effects model, otherwise employing the random effects model. The potential publication bias was assessed by a funnel plot, and Egger's test (39). *P* < 0.05 was considered significant. Statistical analysis was carried out using the “metafor” and “meta” packages of the R/BioConductor (version 3.5.1).

## Results

### Search Results

**Figure 1:**
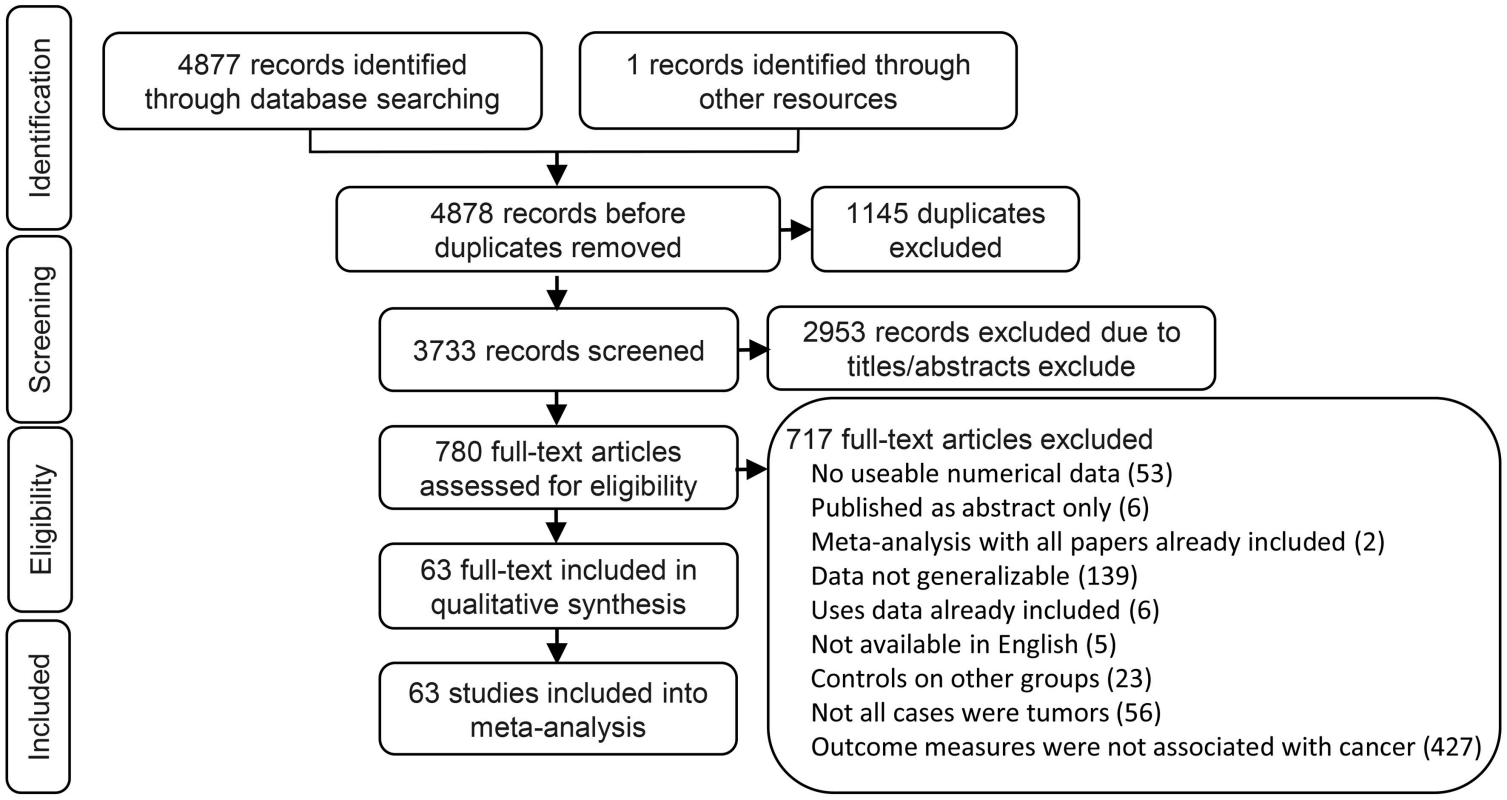
Flow chart of the identification process for eligible studies.

We found 2,397 articles in PubMed, 2,460 articles in EMBASE, 20 articles in Cochrane library, and one articles through the references. We had a total of 3,733 articles after removing 1,145 duplicated articles. We then ruled out 2,953 articles which were review, letters, laboratory studies, or articles irrelevant to present research. We further excluded 717 full-text articles according to the inclusion and exclusion criteria of this study. The remaining 63 articles were finally eligible and included in this meta-analysis (Figure 1).

### Study Demographics

The 63 eligible articles were published in between 2008 and 2017 with 63 studies that included a total of 13,138 participants from 9 countries who represented 16 cancer types and Asian and Caucasian ethnic groups (Table 1). The mean and median value were selected as the cut-off value in most articles. All studies measured SIRT1 expression in tumor tissue or serum via q-PCR, IHC, or ISH.

**Table 1:** Main characteristics of 63 included studies.

**References**	**Study design**	**Country**	**Case (N)**	**Type of Cancer**	**Disease stage**	**Follow-up (Mo.) median(range)**	**Race**	**Sample type**	**Survival end points**	**Adjusted variables**	**NOS score**
Stenzinger et al. (40)	Retrospective	Germany	129	Pancreatic ductal adenocarcinoma	I–IV	54	Caucasian	Tissue	OS	Stage, grade	7
Li et al. (41)	Retrospective	China	91	Pancreatic ductal adenocarcinoma	I–IV	NA	Asian	Tissue	NA	NA	8
Teramae et al. (42)	Retrospective	Japan	62	Uterine cervical cancer	III	NA	Asian	Tissue	OS	NA	7
Asaka et al. (43)	Retrospective	Japan	108	Endometrial carcinoma	I–IV	NA	Asian	Tissue	OS, DFS	NA	8
Jang et al. (44)	Retrospective	Korea	64	Ovarian cancer	I–IV	29 (1–137)	Asian	Tissue	OS	Age, P53	6
Shuang et al. (18)	Retrospective	China	63	Ovarian cancer	I–IV	NA	Asian	Serum	OS	Age, grade, FIGO stage, lymphatic metastasis	6
Mvunta et al. (45)	Retrospective	Japan	68	Ovarian cancer	I–IV	74	Asian	Tissue	OS	Stage, lymphovascular space invasion	7
Zhang et al. (46)	Retrospective	China	22	Osteosarcoma	NA	NA	Asian	Tissue	OS	NA	7
Feng et al. (47)	Retrospective	China	89	Osteosarcoma	I–III	11 (3–83)	Asian	Tissue	OS	Stage, neoadjuvant chemotherapy	8
Kim et al. (24)	Retrospective	Korea	104	Soft tissue sarcoma	I–IV	NA	Asian	Tissue	OS, EFS	P53, β-catenin, stage, depth of tumor, tumor necrosis, distant metastasis	8
Noguchi et al. (29)	Retrospective	Japan	437	Head and neck squamous cell carcinoma	I–IV	46.6 (1–174)	Asian	Tissue	RFS, CSS	Age, gender, tumor site, tumor status, lymph node status, distant metastasis, pathologic grade	8
Yu et al. (48)	Retrospective	China	120	Laryngeal and hypopharyngeal carcinomas	I–IV	NA	Asian	Tissue	OS	NA	7
Batra et al. (49)	Retrospective	India	94	Retinoblastoma	I–IV	59	Asian	Tissue	NA	NA	7
Chen et al. (50)	Retrospective	China	206	Esophageal squamous cell carcinoma	I–II	55.9 (5–86)	Asian	Tissue	OS, DFS	Differentiation, T status, stage, VEGF-C, peritumoral lymphatic microvessel density	7
He et al. (51)	Retrospective	China	86	Esophageal squamous cell carcinoma	I–III	NA	Asian	Tissue	OS	Gender, clinical stage, histological grade, lymph node metastasis	7
Feng et al. (52)	Retrospective	China	34	Pelvis chondrosarcoma	I–III	37.7 vs. 53.8^*^	Asian	Tissue	OS	NA	8
Noh et al. (53)	Retrospective	Korea	200	Renal cell carcinoma	I–III	NA	Asian	Tissue	OS, RFS, CSS	NA	6
Jeh et al. (54)	Retrospective	Korea	102	Renal cell carcinoma	I–IV	73	Asian	Tissue	CSS	Age, T stage, grade, metastasis, karnofsky performance status	7
Jang et al. (28)	Retrospective	Korea	104	Diffuse large B cell lymphoma	I–IV	17.3 (1–135)	Asian	Tissue	OS, EFS	International prognostic index	8
Ren et al. (55)	Retrospective	China	45	Angioimmunoblastic T cell lymphoma	I–IV	NA	Asian	Tissue	PFS	P53, LDH, hemoglobin, γ-Globulin, sex, age, international prognostic index score, stage	8
Nosho et al. (56)	Retrospective	USA	485	Colorectal cancer	I–IV	NA	Caucasian	Tissue	OS, CSS	Age, year of diagnosis, sex, body Mass index, tumor location, stage, grade, microsatellite instability, the CpG island methylator phenotype	6
Jang et al. (2)	Retrospective	Korea	497	Colorectal cancer	I–IV	70.8	Asian	Tissue	OS, DFS	Histological grade, AJCC stage	8
Jung et al. (30)	Retrospective	Korea	349	Colorectal cancer	I–IV	55.3	Asian	Tissue	OS	Age, location, TNM stage, Histologic grade, β-catenin	8
Benard et al. (57)	Retrospective	Netherlands	254	Colorectal cancer	I–III	103.2	Caucasian	Tissue	OS	NA	7
Chen et al. (22)	Retrospective	China	102	Colorectal cancer	II–IV	NA	Asian	Tissue	OS	Gender, age, metastasis, TNM stage	8
Lv et al. (58)	Retrospective	China	120	Colorectal cancer	I–IV	53.3 (1–78)	Asian	Tissue	OS	NA	6
Lee et al. (59)	Retrospective	China	351	Colorectal cancer	I–IV	NA	Asian	Tissue	DFS, CSS	NA	7
Cheng et al. (60)	Retrospective	China	90	Colorectal cancer	I–III	NA	Asian	Tissue	OS	NA	8
Chen et al. (61)	Retrospective	China	172	Hepatocellular Carcinoma	I–III	125 (45–236)	Asian	Tissue	OS	NA	6
Jang et al. (62)	Retrospective	Korea	154	Hepatocellular Carcinoma	I–IV	NA	Asian	Tissue	OS, DFS	Stage, albumin, AFP, c-Myc, P53	8
Hao et al. (63)	Retrospective	China	99	Hepatocellular Carcinoma	I–IV	NA	Asian	Tissue	OS	NA	7
Cheng et al. (64)	Retrospective	China	148	Hepatocellular Carcinoma	I–III	NA	Asian	Tissue	OS	NA	8
Li et al. (65)	Retrospective	China	72	Hepatocellular Carcinoma	I–III	NA	Asian	Tissue	OS, DFS	NA	8
Liu et al. (66)	Retrospective	China	148	Hepatocellular Carcinoma	I–III	NA	Asian	Tissue	NA	NA	7
Cha et al. (27)	Retrospective	Korea	177	Gastric Cancer	I–IV	NA	Asian	Tissue	OS, RFS	TNM stage	7
Feng et al. (67)	Retrospective	China	176	Gastric Cancer	I–IV	NA	Asian	Tissue	OS	NA	6
Kang et al. (68)	Retrospective	Korea	452	Gastric Cancer	I–IV	53.3 (3–83)	Asian	Tissue	OS	Lymph node metastasis, depth of invasion, lymphatic invasion, histologic grade, DBC1, cytoplasmic β-catenin	7
Noguchi et al. (19)	Retrospective	Japan	557	Gastric Cancer	I–IV	69 (6–142)	Asian	Tissue	CSS	NA	7
Qiu et al. (69)	Retrospective	China	96	Gastric Cancer	I–IV	31.6 (6–78)	Asian	Tissue	OS, RFS	Lymph node metastasis, Beclin1 expression	8
Szász et al. (70)	Retrospective	Hungary	1065	Gastric Cancer	I–IV	NA	Caucasian	Tissue	OS	NA	8
Zhang et al. (71)	Retrospective	China	128	Gastric Cancer	I–IV	NA	Asian	Tissue	OS	NA	8
Zhang et al. (72)	Retrospective	China	176	Gastric Cancer	I–IV	NA	Asian	Tissue	OS	NA	6
Ren et al. (73)	Retrospective	USA	348	Colorectal cancer	I–IV	NA	Caucasian	Tissue	OS	NA	8
Shin et al. (74)	Retrospective	Korea	45	Ovarian cancer	NA	NA	Asian	Tissue	OS	NA	6
Zhang et al. (75)	Retrospective	China	50	Colorectal cancer	NA	NA	Asian	Tissue	NA	NA	7
Cao et al. (76)	Retrospective	China	150	Breast carcinoma	I–IV	161	Asian	Tissue	OS, DFS	Lymph node metastasis, TNM stage, ER status, PR status, Snail expression	8
Jin et al. (77)	Retrospective	Korea	319	Breast carcinoma	I–III	NA	Asian	Tissue	OS, DFS	AJCC stage, lymphatic invasion, DCIS	6
Kim et al. (78)	Retrospective	Korea	278	Breast carcinoma	NA	63.78 (2–74)	Asian	Tissue	DFS	T stage, caspase3, lymphovascular invasion	7
Lee et al. (21)	Retrospective	USA	142	Breast carcinoma	I–IV	NA	Caucasian	Tissue	OS, EFS	Stage, HER2 status, P53 expression	8
Chung et al. (79)	Retrospective	Korea	344	Breast carcinoma	I–III	NA	Asian	Tissue	OS, DFS	T stage, lymphatic invasion, DCIS	6
Derr et al. (3)	Retrospective	Netherlands	822	Breast carcinoma	I–III	11.8 (0.16–27.55)	Caucasian	Tissue	OS, DFS	NA	6
Wu et al. (25)	Retrospective	China	134	Breast carcinoma	I–III	154	Asian	Tissue	OS, DFS	Stages, P53,Lymph nodes status	8
Lee et al. (80)	Retrospective	Korea	688	Breast carcinoma	I–IV	190.8	Asian	Tissue	NA	NA	8
Chung et al. (81)	Retrospective	Korea	427	Breast carcinoma	I–III	NA	Asian	Tissue	DFS	NA	6
Zhang et al. (82)	Retrospective	China	149	Breast carcinoma	NA	101.03 vs. 88.38^*^	Asian	Tissue	OS	NA	6
Sung et al. (83)	Retrospective	Korea	28	Breast carcinoma	I-IV	NA	Asian	Tissue	NA	NA	6
Gharabaghi et al. (16)	Retrospective	Iran	40	NSCLC	NA	NA	Caucasian	Tissue	OS	Gender, age, histologic grade, T stage, lymph node metastasis, BIRC6 expression	6
Li et al. (20)	Retrospective	China	75	NSCLC	I–IV	NA	Asian	Tissue	OS	Age, TNM stage	7
Lin et al. (84)	Retrospective	China	260	NSCLC	NA	37.1 (0–128)	Asian	Tissue	OS	NA	8
Noh et al. (85)	Retrospective	Korea	144	NSCLC	NA	NA	Asian	Tissue	NA	NA	7
Zhang et al. (86)	Retrospective	China	295	NSCLC	III–IV	NA	Asian	Tissue	OS	Tumor stage, tumor differentiation	6
Chen et al. (23)	Retrospective	China	125	NSCLC	I–IV	NA	Asian	Tissue	NA	NA	8
Grbesa et al. (87)	Retrospective	Spain	179	NSCLC	I–IV	45	Asian	Tissue	OS, RFS	Stage	6

Mo., month; NSCLC, non-small cell lung cancer; NA, not available; NOS, newcastle-ottawa scale. FIGO, the international federation of gynecology and obstetrics; OS, overall survival; EFS, event-free survival; DFS, disease-free survival; CSS, cancer-specific survival; RFS, recurrence-free survival; LDH, lactate dehydrogenase; DBC1, deleted in breast cancer 1; ER, estrogen receptor; PR, progesterone receptor; DCIS, ductal carcinoma in situ; HER2, human epidermal growth factor receptor 2; BIRC6, Baculoviral IAP repeat-containing 6.

* *The median survival time of high expression group vs. low expression group*.

### Correlation Between SIRT1 Expression and Prognosis

**Figure 2:**
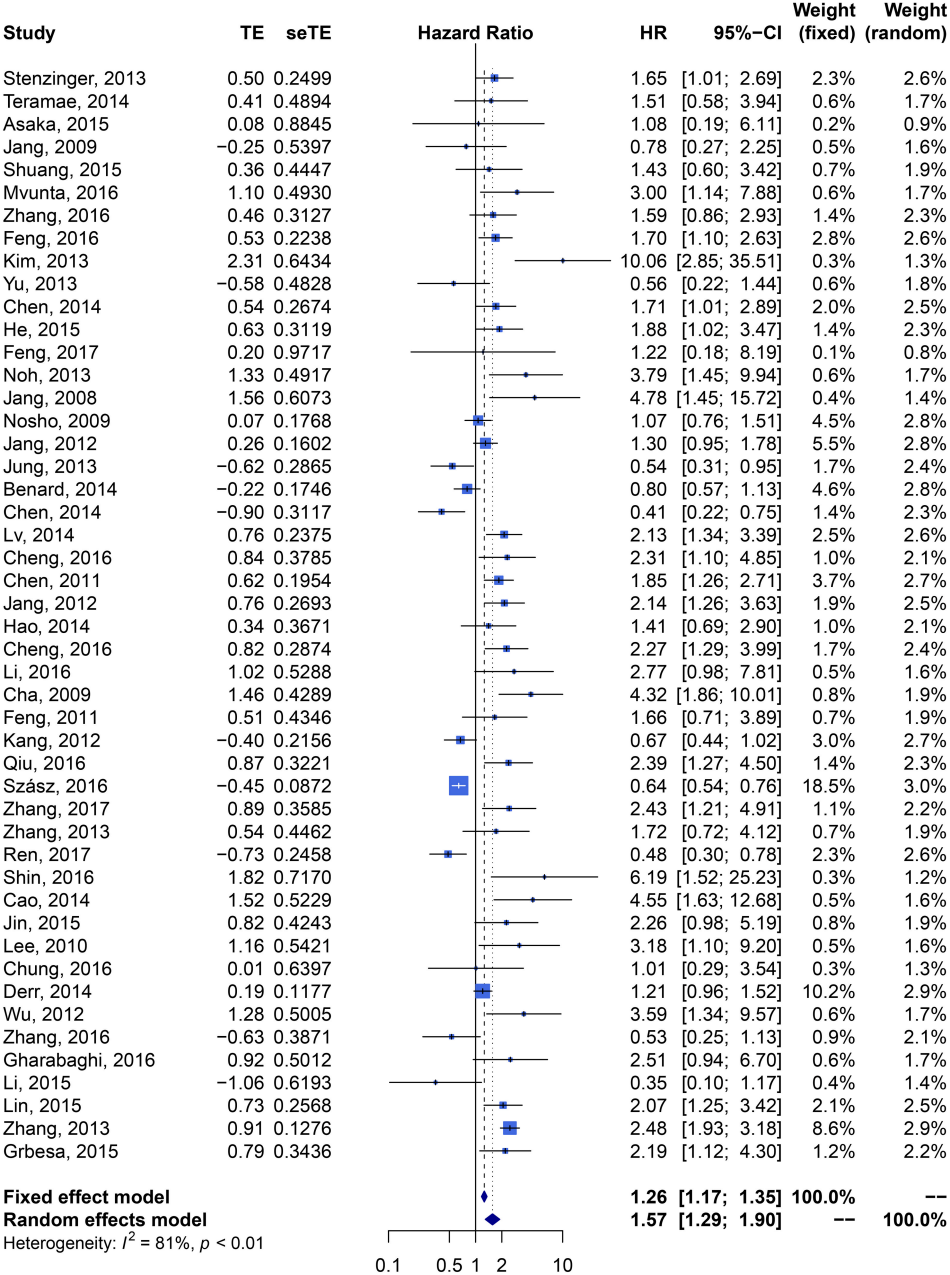
Forest plot of SIRT1 expression and overall survival in various cancers.

**Figure 3:**
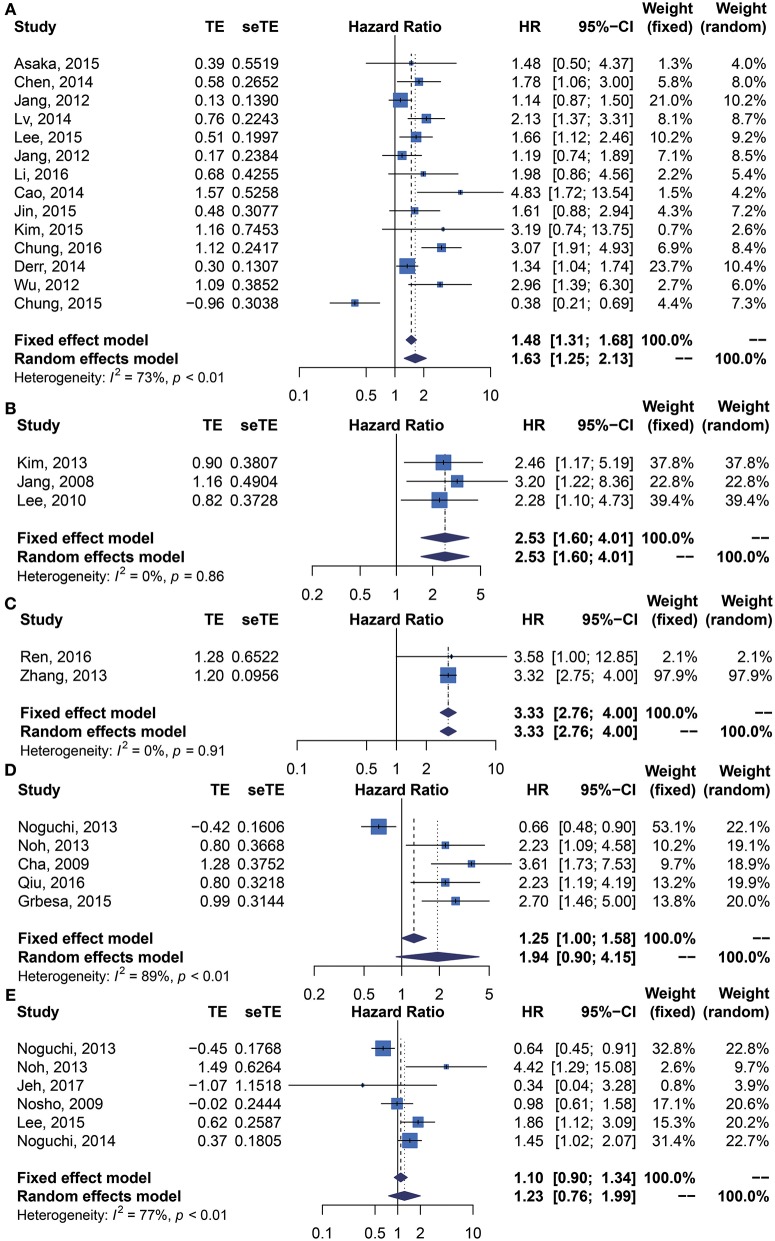
Forest plots of main survival outcomes compared SIRT1 overexpression with underexpression. **(A)** DFS. **(B)** EFS. **(C)** PFS. **(D)** RFS. **(E)** CCS.

We performed meta-analysis of correlation between SIRT1 expression and OS, DFS, EFS, RFS, CCS, and PFS. The results and analysis of publication bias are presented in Table 2. The results showed that higher SIRT1 expression indicated an unfavorable OS (*n* = 48, HR: 1.566, 95% CI: [1.293, 1.895], *P* < 0.0001, *I*^2^ = 81.3%) (Figure 2), poor patient DFS (*n* = 14, HR: 1.631, 95% CI: [1.250–2.130], *P* = 0.0003, *I*^2^ = 72.6%, Figure 3A), poor EFS (*n* = 3, HR: 2.534, 95% CI: [1.602, 4.009], *P* = 0.0001, *I*^2^ = 0%, Figure 3B), and poor PFS (*n* = 2, HR: 3.325, 95% CI: [2.762, 4.003], *P* < 0.0001, *I*^2^ = 0%, Figure 3C), but not correlated with RFS of the Asian or tissue (*n* = 5, HR: 1.936, 95% CI: [0.903 - 4.151], *P* = 0.0898, *I*^2^ = 88.9%) (Figure 3D) or CCS (*n* = 6, HR: 1.229, 95% CI: [0.757–1.994], *P* = 0.4037, *I*^2^ = 77.3%) (Figure 3E).

**Table 2:** Survival effects of SIRT1 overexpression and the prognosis of patients.

**Outcome**	**No. of trials (patients)**	**HR (95%CI) Fixed-effect estimate**	***P*-value of Fixed-effect Model**	**HR (95%CI) Random-effect estimate**	***P* value of Random-effect Model**	**Heterogeneity *I*^**2**^(%), *P*-value**	***P*-value of Egger's test, Begg's test**
OS	48 (9573)	1.259 (1.170–1.355)	< 0.0001	***1.566 (1.293–1.895)***	*** < 0.0001***	81.3%, < 0.0001	0.0043, 0.1884
DFS	14 (3982)	1.482 (1.308–1.679)	< 0.0001	***1.631 (1.250–2.130)***	***0.0003***	72.6%, < 0.0001	0.2234, 0.2503
EFS	3 (350)	***2.534 (1.602–4.009)***	***0.0001***	2.534 (1.602–4.009)	0.0001	0.0%, 0.8557	0.1174, 0.1172
RFS	5 (1089)	1.253 (0.996–1.575)	0.0542	1.936 (0.903–4.151)	0.0898	88.90%, < 0.0001	0.0037, 0.3272
CCS	6 (2132)	1.097 (0.900–1.338)	0.3591	1.229 (0.757–1.994)	0.4037	77.3%, 0.0005	0.6331, 0.3476
PFS	2 (340)	***3.325 (2.762–4.003)***	*** < 0.0001***	3.325 (2.762–4.003)	< 0.0001	0.0%, 0.9089	NA, NA

*HR, hazard ratio; CI, confidence interval; OS, overall survival; DFS, disease free survival; EFS, event free survival; RFS, recurrence-free survival; CCS, cancer-specific survival; PFS, progress-free survival; NA, not available. I^2^, index for assessing heterogeneity; value ≥25% indicates a moderate to high heterogeneity; Egger's test, P-value of Egger's regression for asymmetry assessment; Begg's test, P-value of Begg and Mazumdar rank correlation test for asymmetry assessment. Bold italics indicate statistically significant values (P < 0.05)*.

### Correlation Between SIRT1 Expression and Prognosis of Cancer Types

Cancer type subgroup analysis showed that SIRT1 overexpression was associated with a worse OS in osteosarcoma (*n* = 2, HR: 1.661, 95% CI: [1.162, 2.372], *P* = 0.0053, *I*^2^ = 0%), esophageal squamous cell carcinoma (*n* = 2, HR: 1.781, 95% CI: [1.197, 2.652], *P* = 0.0044, *I*^2^ = 0%), hepatocellular carcinoma (*n* = 5, HR: 1.969, 95% CI: [1.539, 2.520], *P* < 0.0001, *I*^2^ = 0%), breast carcinoma (*n* = 7, HR: 1.744, 95% CI: [1.022, 2.978], *P* < 0.0416, *I*^2^ = 70.18%), NSCLC (*n* = 5, HR: 1.929, 95% CI: [1.259, 2.957], *P* < 0.0025, *I*^2^ = 59.40%), whereas SIRT1 overexpression was not correlated with the OS in ovarian cancer (*n* = 4, HR: 1.971, 95% CI: [0.899, 4.323], *P* = 0.0903, *I*^2^ = 55.18%), colorectal carcinoma (*n* = 8, HR: 0.932, 95% CI: [0.636, 1.366], *P* = 0.7198, *I*^2^ = 82.96%), gastric carcinoma (*n* = 7, HR: 1.535, 95% CI: [0.864, 2.726], *P* = 0.1436, *I*^2^ = 87.90%) (Supplementary Figure 1A).

SIRT1 overexpression was associated with a worse DFS in colorectal cancer (*n* = 3, HR: 1.544, 95% CI: [1.061, 2.247], *P* = 0.0233, *I*^2^ = 68.69%), and breast carcinoma (*n* = 7, HR: 1.819, 95% CI: [1.026, 3.223], *P* = 0.0404, *I*^2^ = 84.59%), whereas it was not correlated with the DFS in hepatocellular carcinoma (*n* = 2, HR: 1.357, 95% CI: [0.872, 2.113], *P* = 0.1758, *I*^2^ = 9.41%) (Supplementary Figure 2A).

SIRT1 overexpression was correlated with RFS of Gastric Cancer (*n* = 2, HR: 2.734, 95% CI: [1.694, 4.413], *P* < 0.0001, *I*^2^ = 0%), Renal cell carcinoma (*n* = 1, HR: 2.233, 95% CI: [1.088, 4.583]), and NSCLC (HR: 2.698, 95% CI: [1.457, 4.996]), whereas SIRT1 overexpression was negatively correlated with RFS of head and neck squamous cell carcinoma (HR: 0.655, 95% CI: [0.478, 0.897], *P* = 0.0084) (Supplementary Table 3).

SIRT1 overexpression was correlated with CCS in head and neck squamous cell carcinoma (*n* = 1, HR: 0.640, 95% CI: [0.453, 0.905], *P* = 0.0116), gastric cancer (*n* = 1, HR: 1.450, 95% CI: [1.018, 2.066], *P* = 0.0396), and renal cell carcinoma (*n* = 2, HR: 1.478, 95% CI: [0.124, 17.621]), but not with CCS in colorectal cancer (HR: 1.344, 95% CI: [0.716, 2.521], *P* = 0.3577) (Supplementary Table 3).

### Correlation Between SIRT1 Expression and Prognosis of Cancer in Different Countries

Analysis of country subgroups showed that high expression of SIRT1 was correlated with poor OS in China (*n* = 24, HR: 1.661, 95% CI: [1.339, 2.060], *P* < 0.0001, *I*^2^ = 63.03%), Korea (*n* = 12, HR: 1.902, 95% CI: [1.187, 3.047], *P* = 0.0075, *I*^2^ = 80.65%), Japan (*n* = 3, HR: 1.940, 95% CI: [1.029, 3.655], *P* = 0.0405, *I*^2^ = 0%), but not in USA (*n* = 3, HR: 1.043, 95% CI: [0.465, 2.338], *P* = 0.9193, *I*^2^ = 84.36%), or Netherlands (*n* = 2, HR: 1.003, 95% CI: [0.671, 1.498], *P* = 0.9893, *I*^2^ = 73.79%) (Supplementary Figure 1B).

SIRT1 overexpression was also correlated with poor DFS in China (*n* = 6, HR: 2.021, 95% CI: [1.612, 2.534], *P* < 0.0001, *I*^2^ = 0%), but not in Korea (*n* = 6, HR: 1.321, 95% CI: [0.773, 2.259]) (Supplementary Figure 2B).

SIRT1 overexpression was correlated with poor EFS in Korea (*n* = 2, HR: 2.714, 95% CI: [1.506, 4.894], *P* = 0.0009, *I*^2^ = 0%) and USA (*n* = 1, HR: 2.280, 95% CI: [1.098, 4.734]).

### Correlation Between SIRT1 Expression and Prognosis of Cancer in Asian and Caucasian

Elevated SIRT1 expression predicted a significantly worse OS in Asian population with cancers (HR: 1.708, 95% CI: [1.406, 2.076], *P* < 0.0001, *I*^2^ = 69.59%) rather than in Caucasian population (HR: 1.04, 95% CI: [0.75, 1.45], *P* < 0.01, *I*^2^ = 81%) (Supplementary Figure 1C).

SIRT1 expression predicted a significantly worse DFS in Asian population with cancers (*n* = 13, HR: 1.683, 95% CI: [1.235, 2.294], *P* < 0.0010, *I*^2^ = 74.27%), whereas one article suggested that increased expression of SIRT1 is correlated with Caucasian patient DFS (HR: 1.344, 95% CI: [1.040; 1.736], *P* = 0.0237) (Supplementary Figure 2C).

### Correlation Between SIRT1 Expression and Clinicopathological Characteristics

**Figure 4:**
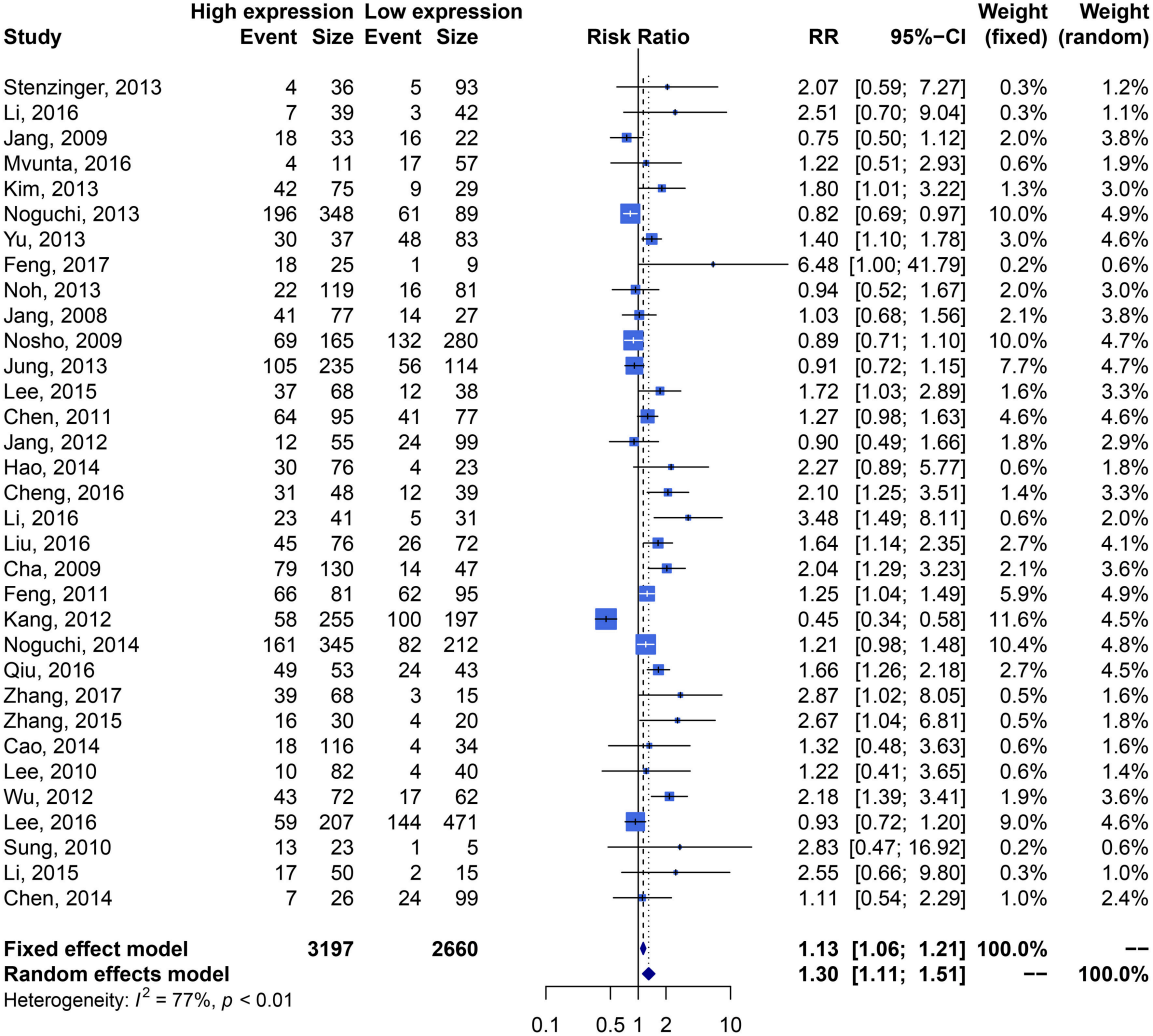
Forest plots of tumor stage compared SIRT1 overexpression with underexpression.

**Figure 5:**
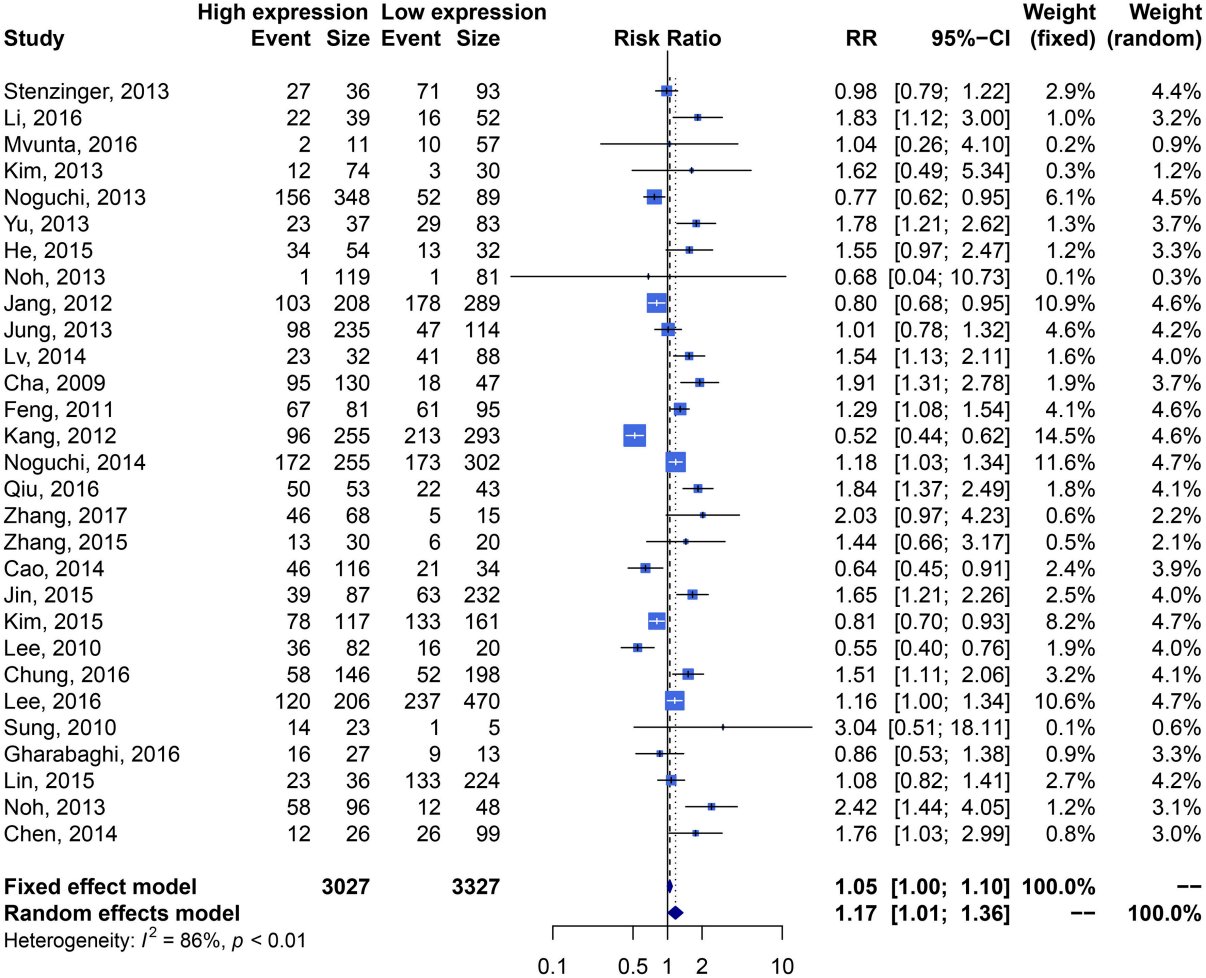
Forest plots of lymphatic metastasis compared SIRT1 overexpression with underexpression.

**Figure 6:**
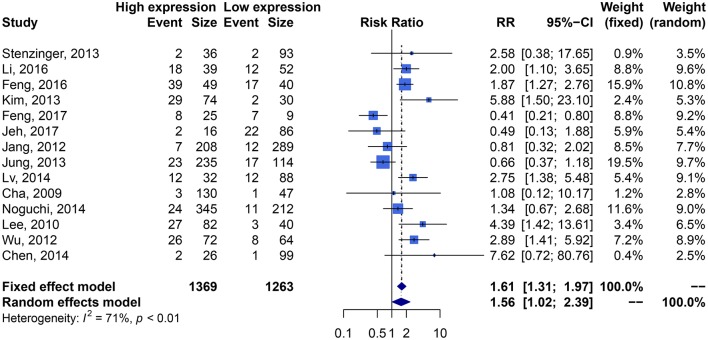
Forest plots of distant metastasis compared SIRT1 overexpression with underexpression.

We performed an analysis of the association of SIRT1 expression with clinicopathological characteristics (Table 3). The results showed that overexpression of SIRT1 was significantly correlated with TNM stage. Higher SIRT1 expression indicated high TNM stage for various malignancies (*n* = 33, RR: 1.299; 95% CI: [1.114, 1.514], *P* = 0.0008, *I*^2^ = 77.4%, Figure 4). SIRT1 expression was significantly correlated with lymphatic metastasis (*n* = 29, RR: 1.172, 95% CI: [1.010, 1.360], *P* = 0.0363, *I*^2^ = 86.3%, Figure 5), distant metastasis (*n* = 14, RR: 1.562, 95% CI: [1.022, 2.387], *P* = 0.0392, *I*^2^ = 71.0%, Figure 6), but not correlated with tumor size (RR:1.101, 95% CI [0.984-1.232], *I*^2^ = 41.7%), depth of tumor invasion (RR: 1.113, 95% CI [0.985–1.258], *I*^2^ = 81.7%), differentiation (RR: 1.055, 95% CI [0.931–1.196], *I*^2^ = 63.1%), gender (RR: 0.991, 95% CI [0.950–1.035], *I*^2^ = 35.0%), or age (RR: 1.043, 95% CI [0.973–1.118], *I*^2^ = 43.5%) (Table 3, Supplementary Figure 3).

**Table 3:** The associations of SIRT1 overexpression with the clinicopathological characteristics of the study patients.

**Clinicopathological parameters**	**No. of trials (patients)**	**RR (95%CI) Fixed-effect estimate**	***P*-value of Fixed-effect Model**	**RR (95%CI) Random-effect estimate**	***P*-value of Random-effect Model**	**Heterogeneity *I*^**2**^(%), *P*-value**	***P*-value of Egger's test, Begg's test**
Tumor stage	33 (5857)	1.133 (1.062–1.209)	0.0002	***1.299 (1.114–1.514)***	***0.0008***	77.4%, < 0.0001	0.0070, 0.1827
Lymphatic metastasis	29 (6354)	1.046 (0.995–1.100)	0.0763	***1.172 (1.010–1.360)***	***0.0363***	86.3%, < 0.0001	0.0637, 0.4308
Distant metastasis	14 (2632)	1.607 (1.312–1.968)	< 0.0001	***1.562 (1.022–2.387)***	***0.0392***	71.0%, < 0.0001	0.6780, 0.3520
Tumor size	21 (2469)	1.143 (1.050–1.245)	0.0021	1.101 (0.984–1.232)	0.0924	41.7%, 0.0241	0.1660, 0.2047
Depth of tumor invasion	19 (4689)	1.036 (0.982–1.093)	0.1912	1.113 (0.985–1.258)	0.0852	81.70%, < 0.0001	0.0903, 0.1955
Differentiation	28 (5740)	1.010 (0.940–1.085)	0.7841	1.055 (0.931–1.196)	0.3996	63.10%, < 0.0001	0.1170, 0.3847
Age	38 (7223)	1.052 (1.004–1.102)	0.0345	1.043 (0.973–1.118)	0.2373	43.50%, 0.0027	0.5651, 0.8308
Gender	34 (6129)	1.003 (0.967–1.040)	0.8739	0.991 (0.950–1.035)	0.6858	35.00%, 0.0247	0.1727, 0.3353

*RR, relative risk; CI, confidence interval; I^2^, index for assessing heterogeneity; value ≥25% indicates a moderate to high heterogeneity; Egger's test, P-value of Egger's regression for asymmetry assessment; Begg's test: P-value of Begg and Mazumdar rank correlation test for asymmetry assessment. Bold italics indicate statistically significant values (P < 0.05)*.

### Correlation Between SIRT1 Expression and Clinicopathological Characteristics of Cancers Types

We performed analysis of correlation between SIRT1 expression and clinicopathological characteristics of cancers types (Supplementary Table 4). The results showed that SIRT1 overexpression was associated with a higher TNM stage in hepatocellular carcinoma (*n* = 6, RR: 1.611, 95% CI: [1.185, 2.188], *P* = 0.0023, *I*^2^ = 55.30%), but not correlated with the TNM stage in pancreatic ductal adenocarcinoma (*n* = 2, RR: 2.275, 95% CI: [0.928, 5.579], *P* = 0.0725, *I*^2^ = 0%), ovarian cancer (*n* = 2, RR: 0.820, 95% CI: [0.561, 1.201], *P* = 0.3082, *I*^2^ = 3.18%), colorectal cancer (*n* = 4, RR: 1.146, 95% CI: [0.817, 1.608], *P* = 0.4290, *I*^2^ = 70.90%), gastric cancer (*n* = 6, RR: 1.264, 95% CI: [0.823, 1.942], *P* = 0.2842, *I*^2^ = 92.48%), breast carcinoma (*n* = 5, RR: 1.411, 95% CI: [0.846, 2.356], *P* = 0.1873, *I*^2^ = 65.10%), or NSCLC (n = 2, RR: 1.389, 95% CI: [0.661, 2.917], *P* = 0.3853, *I*^2^ = 16.51%) (Supplementary Figure 4A).

SIRT1 overexpression was associated with distant metastasis in pancreatic ductal adenocarcinoma (*n* = 2, RR: 2.046, 95% CI: [1.153, 3.631], *P* = 0.0144, *I*^2^ = 0%) and breast carcinoma (*n* = 2, RR: 3.257, 95% CI: [1.777, 5.970], *P* = 0.0001, *I*^2^ = 0%), but not in colorectal cancer (*n* = 3, RR: 1.140, 95% CI: [0.444, 2.923], *P* = 0.7857, *I*^2^ = 80.57%) or gastric cancer (*n* = 2, RR: 1.316, 95% CI: [0.679, 2.551], *P* = 0.4160, *I*^2^ = 0%) (Supplementary Figure 5A).

### Correlation Between SIRT1 Expression and Clinicopathological Characteristics of Cancers in Different Countries

We performed analysis of correlation between SIRT1 expression and clinicopathological characteristics of cancers in different countries (Supplementary Table 4). The results showed that SIRT1 overexpression was associated with a higher TNM stage (*n* = 17, RR: 1.638, 95% CI: [1.404, 1.911], *P* < 0.0001, *I*^2^ = 41.16%) (Supplementary Figure 4B) and lymphatic metastasis in China (*n* = 11, RR: 1.411, 95% CI: [1.155, 1.724], *P* = 0.0007, *I*^2^ = 68.48%), and not with lymphatic metastasis in Japan (*n* = 3, RR: 0.964, 95% CI: [0.657, 1.415]), or Korea (*n* = 12, RR: 1.166, 95% CI: [0.898, 1.516]) (Supplementary Figure 6A).

### Correlation Between SIRT1 Expression and Clinicopathological Characteristics of Cancers in Asian and Caucasian

We performed analysis of correlation between SIRT1 expression and clinicopathological characteristics of cancers in Asian and Caucasian (Supplementary Table 4). The results showed that SIRT1 overexpression predicted a significantly higher TNM stage in Asian population with cancers (*n* = 30, RR: 1.323, 95% CI: [1.124, 1.559], *P* = 0.0008, *I*^2^ = 78.76%) rather than that in Caucasian population (*n* = 3, RR: 0.919, 95% CI: [0.744, 1.136], *P* = 0.4352, *I*^2^ = 0%) (Supplementary Figure 4C). However, publication bias was suspected based on the Egger's test (*P* = 0.0070) rather than Begg's test (*P* = 0.1827).

Elevated SIRT1 expression predicted a significantly distant metastasis in Caucasian population with cancers (*n* = 2, RR: 3.830, 95% CI: [1.445, 10.154], *P* = 0.0069, *I*^2^ = 0%), but not in Asian population (*n* = 12, RR: 1.422, 95% CI: [0.913, 2.217], *P* = 0.1198, *I*^2^ = 72.85%) (Supplementary Figure 5B).

Elevated SIRT1 expression predicted a significantly higher lymphatic metastasis in Asian population with cancers (*n* = 29, RR: 1.239, 95% CI: [1.056, 1.453], *P* = 0.0086, *I*^2^ = 86.81%), but not correlated with lymphatic metastasis in Caucasian population (*n* = 3, RR: 0.777, 95% CI: [0.526, 1.147], *P* = 0.2040, *I*^2^ = 76.11%) (Supplementary Figure 6B).

### Meta-Regression Analysis of Heterogeneity for Overall Survival and Publication Bias

We performed a meta-regression to explore the source of high heterogeneity for OS (Table 4). All potential factors could not significantly explain heterogeneity in the meta-analyses of the SIRT1 expression with survival outcomes in the *post-hoc* analysis, with the exception of ethnicity (Supplementary Table 5). Meta-regression analysis demonstrated a statistically significant correlation between ethnicity and OS (*P* = 0.022). From the meta-regression result, we conducted a subgroup analysis with groups of patients Asian or Caucasian (Supplementary Figure 1C). This subgroup analysis demonstrated a significantly lower heterogeneity value in Asian group (*n* = 40, RR: 1.708, 95% CI: [1.406, 2.076], *I*^2^ = 69.59%), which suggests that the high SIRT1 expression has stronger efficacy in the Asian population than the Caucasian population.

**Table 4:** Meta-regression analysis of heterogeneity for overall survival.

**Moderators**	**Variables of regression**	**HR_**interaction**_ (95% CI)**	***P*-value of regression**	***I*^**2**^**	**Cochrane Q (*P*-value)**
Year	Year	1.001(0.990–1.012)	0.840	81.69%	< 0.001
Sample size	Sample size	2.578(0.674–9.860)	0.166	43.18%	0.152
Follow up	Follow up	0.741(0.076–7.236)	0.796	72.89%	< 0.001
Country	Intercept	1.648(1.291–2.104)	< 0.001	71.56%	< 0.001
	Germany	1.001(0.334–2.997)	0.999	71.56%	< 0.001
	Hungary	0.388(0.143–1.051)	0.063	71.56%	< 0.001
	Iran	1.523(0.380–6.105)	0.553	71.56%	< 0.001
	Japan	1.134(0.463–2.775)	0.783	71.56%	< 0.001
	Korea	1.079(0.695–1.675)	0.736	71.56%	< 0.001
	Netherlands	0.600(0.285–1.263)	0.178	71.56%	< 0.001
	Spain	1.330(0.405–4.374)	0.638	71.56%	< 0.001
	USA	0.605(0.302–1.215)	0.158	71.56%	< 0.001
Tumor type	Intercept	1.716(1.055–2.792)	0.030	75.22%	< 0.001
	Colorectal cancer	0.543(0.293–1.008)	0.053	75.22%	< 0.001
	Diffuse large B cell lymphoma	2.786(0.559–13.892)	0.211	75.22%	< 0.001
	Endometrial carcinoma	0.629(0.082–4.849)	0.657	75.22%	< 0.001
	Esophageal squamous cell carcinoma	1.044(0.412–2.640)	0.928	75.22%	< 0.001
	Gastric Cancer	0.851(0.442–1.638)	0.630	75.22%	< 0.001
	Hepatocellular Carcinoma	1.168(0.574–2.373)	0.668	75.22%	< 0.001
	Laryngeal and hypopharyngeal carcinomas	0.326(0.078–1.370)	0.126	75.22%	< 0.001
	NSCLC	1.071(0.521–2.204)	0.852	75.22%	< 0.001
	Osteosarcoma	0.960(0.384–2.399)	0.930	75.22%	< 0.001
	Ovarian cancer	1.135(0.478–2.692)	0.774	75.22%	< 0.001
	Pancreatic ductal adenocarcinoma	0.961(0.294–3.144)	0.948	75.22%	< 0.001
	Pelvis chondrosarcoma	0.711(0.080–6.345)	0.760	75.22%	< 0.001
	Renal cell carcinoma	2.208(0.520–9.383)	0.283	75.22%	< 0.001
	Soft tissue sarcoma	***5.863(1.115–30.823)***	***0.037***	75.22%	< 0.001
	Uterine cervical cancer	0.880(0.208–3.727)	0.862	75.22%	< 0.001
Race	Intercept	1.705(1.414–2.056)	< 0.001	73.32%	< 0.001
	Caucasian	***0.619(0.411–0.932)***	***0.022***	73.32%	< 0.001
Sample type	Intercept	1.430(0.353–5.799)	0.617	81.72%	< 0.001
	Tissue	1.097(0.267–4.510)	0.898	81.72%	< 0.001

*HR_interaction_, interaction effect calculated by meta-regression; Positive direction indicates that possible moderators might strengthen OS in the SIRT1 overexpression relative to underexpression. Bold italics indicate statistically significant values (P < 0.05)*.

Meta-regression also used to explore the source of high heterogeneity for clinicopathological outcomes (Further details are provided in Supplementary Table 6). As to tumor stage, meta-regression analysis demonstrated a statistically significant correlation between tumor stage and country (*P* < 0.05), published year (*P* = 0.0169), and sample size (*P* = 0.0004). This subgroup analysis demonstrated a significantly lower heterogeneity value in China (*n* = 17, RR: 1.638, 95% CI: [1.404, 1.911], *I*^2^ = 41.16%), which suggests that the high SIRT1 expression has stronger efficacy in the China population than the other countries. As to tumor size, meta-regression analysis demonstrated a statistically significant correlation between tumor size and published year (*P* = 0.0260). As to depth of tumor invasion, meta-regression analysis demonstrated a statistically significant correlation between depth of tumor invasion and sample size (*P* = 0.0044).

**Figure 7:**
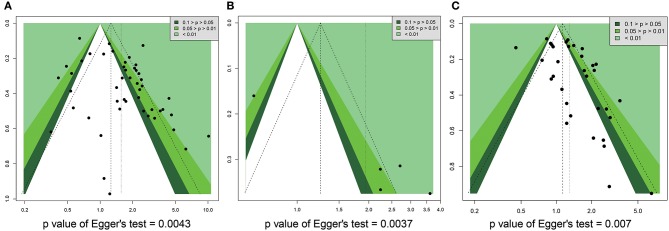
Funnel plot for publication bias in overall survival, recurrence free survival, and TNM stage. **(A)** OS. **(B)** RFS. **(C)** TNM stage.

We used funnel plots and Egger's regression models to assess potential publication bias (Tables 2, 3). The association between HRs (Supplementary Figure 7) or RRs (Supplementary Figure 8) and standard error for the SIRT1 expression was demonstrated in funnel plots, with each plot point representing a study. In regards to the OS, RFS, and TNM stage, we found that Egger's regression yielded potential publication bias (Figure 7).

## Discussion

In the current study, we conducted a meta-analysis of 13,138 subjects in 63 articles from PubMed, EMBASE and Cochrane library to evaluate prognostic and clinicopathological significance of SIRT1 expression in cancers. We found that elevated expression of SIRT1 was correlated with a poor OS of cancer patients, DFS, EFS, and PFS, but couldn't predict RFS or CCS. Elevated SIRT1 expression was associated with TNM stage, lymph node metastasis, and distant metastasis, but not with tumor size, depth of tumor invasion, differentiation, gender, or age. Our findings provide a clue to understanding prognostic and clinicopathological significance of SIRT1 expression in cancers.

Our current study indicates that overexpression of SIRT1 is correlated with poor OS, DFS, EFS, and PFS, but not with RFS or CCS, suggesting that SIRT1 expression is significantly correlated with poor prognosis as a global factor but not a restricted factor to tumor itself. It has been shown that SIRT1 is not a protein only found to a specific tissues or organs, instead, its expression can be found in almost all human tissues (1) and involved in a large variety of cellular processes, such as genomic stability, energy metabolism, senescence, gene transcription, and oxidative stress (5) by acting on a wide spectrum of proteins, including histones and transcription factors (2–4). This render SIRT1 plays multiple roles to regulate biological process in multi-systems. Melatonin is a pleiotropic molecule synthesized by pineal gland and many other organs and has important cytoprotective effects in many tissues including aging, neurodegenerative diseases, immunomodulation, and cancer and modulates DNA damage response (88, 89). Melatonin counteracts tumor metastases by modulating cell-cell and cell-matrix interaction, extracellular matrix remodeling, cytoskeleton reorganization, epithelial-mesenchymal transition, and angiogenesis (90). Recent studies showed that the upregulated SIRT1 signaling pathway is involved in protective effects of melatonin on vascular endothelium against aging-, oxidative stress-, lipopolysaccharide-, and ischemia-induced damage (91) and delays ovarian aging (92). SIRT1 is induced in normal cells and inhibited in tumor cells by melatonin (88, 89). SIRT1 may mediate the pleiotropic function of melatonin in cancer progression and metastasis. SIRT1 is an endocrine regulator of thyroid and parathyroid hormone function (93–95), and steroid hormone receptor activity (96, 97). SIRT1 is a regulator in immunity and autoimmunity, such as dendritic cell activation, T-regulatory cells (98–102). SIRT1 is also a regulator of lipid and carbohydrate metabolism (9, 103–105). In addition, SIRT1 regulates nervous system by inhibiting neuronal apoptosis and damage as well module nerve regeneration (103, 106, 107). Therefore, SIRT1 is a global factor for endocrine, immunity, metabolism, and nervous system, and affect poor OS, DFS, EFS, and PFS, but not with RFS or CCS in cancer patients.

In the current study, we found that SIRT1 overexpression was associated with TNM stage, lymph node metastasis, and distant metastasis, but not with tumor size, depth of tumor invasion, differentiation, gender, or age, suggesting that SIRT1 promotes metastasis but not growth, proliferation, and invasion of cancer tissues. Tumor is locally initiated and proliferated and may invade near tissues. Tumor size, depth of tumor invasion, and differentiation are terms used to characterize tumors which are locally confined in the early stage of malignancy (108, 109). Metastasis is the characteristics of advanced malignancy of cancer progression (110–113). Our data indicate that SIRT1 overexpression is associated with metastasis but not with tumor characteristics of early stage, suggesting that SIRT1 expression can predict advanced malignancy and is a potential therapeutic target for inhibiting metastasis of advanced cancer.

We performed subgroup analysis because of high heterogeneity in included studies. Correlation analysis between SIRT1 expression and prognosis of cancer types showed that SIRT1 overexpression predicted worse OS of osteosarcoma, esophageal squamous cell carcinoma, OS but not DFS of hepatocellular carcinoma, OS and DFS of breast carcinoma, OS and RFS of NSCLC, DFS but not OS or CCS in colorectal cancer, RFS and CCS but not OS of gastric cancer, RFS and CCS of renal cell carcinoma, CCS but not RFS in head and neck squamous cell carcinoma. SIRT1 overexpression cannot predict OS in ovarian cancer. Correlation analysis between SIRT1 expression and clinicopathological characteristics of cancers types showed that SIRT1 overexpression was associated with a higher TNM stage in hepatocellular carcinoma, but not in pancreatic ductal adenocarcinoma, ovarian cancer, colorectal cancer, gastric cancer, breast carcinoma, or NSCLC. SIRT1 overexpression was associated with distant metastasis in pancreatic ductal adenocarcinoma and breast carcinoma, but not in colorectal cancer or gastric cancer. From these results, we are unable to draw a unanimous conclusion, probably because there is a deficiency of studies that employ all prognostic indexes OS, DFS, EFS, RFS, CCS, and PFS or a full range of clinicopathological characteristics to study the role of SIRT1 expression in survival of patients with a specific cancer type. More thorough studies are warranted.

Our subgroup correlation analysis between SIRT1 expression and prognosis of cancer in different countries and ethnic groups showed that high expression of SIRT1 predicted poor OS and DFS in China, poor OS and EFS but not DFS in Korea, poor OS in Japan, EFS in USA, but not OS in USA or Netherlands. Elevated SIRT1 expression predicted worse OS and DFS in Asian population with cancers, poor DFS but not OS in Caucasian population. Our subgroup analysis between SIRT1 expression and clinicopathological characteristics of cancers in different countries and ethnic groups showed that SIRT1 overexpression was associated with a higher TNM stage and lymphatic metastasis in China and Asian population except lymphatic metastasis in Japan or Korea, and not higher TNM stage and lymphatic metastasis in Caucasian population, We also found that SIRT1 overexpression predicted distant metastasis in Caucasian population, but not in Asians. These results indicate that ethnic background has influence on the role of SIRT1 expression in predicting the OS and clinicopathological characteristics of cancers. This is consistent with recent studies that showed SIRT1 expression is lower in NSCLC than the normal control group in a group of Chinese patients (15), and overexpressed in NSCLC in an Iran population (16). Our study showed that overexpression of SIRT1 predicted a worse OS in the Asian but not in the Caucasian, a higher TNM stage and lymphatic metastasis in Asian population especially in China but not in the Caucasian. This is consistent with the results of our meta-regression analysis. The effects of ethnic background on the role of SIRT1 expression in predicting the OS and clinicopathological characteristics of cancers need further collaborative investigation.

It has been established that there are significant differences between Asian and Caucasian populations in genetic and epigenetic background, dietary, environmental factors (114, 115). These factors are essential for not only initiation and progression, but also metastasis of cancers (116, 117). Mutations and extensive polymorphisms of SIRT1 were found in Chinese and Japanese (118–121) and 41 cancer lines (122). Although the data on mutations and polymorphisms of SIRT1 are very limited, we speculate that difference in SIRT1 mutations and polymorphisms may be one of accounts for difference in predicting OS and TNM stage and lymphatic metastasis of cancer by SIRT1 expression. This deserves further investigation (123).

It is known that metastasis is an independent predictor for poor prognosis of many cancer types (124–126). We find that elevated expression of SIRT1 was correlated with OS, DFS, EFS, and PFS. SIRT1 overexpression is also correlated with TNM stage, lymph node metastasis, and distant metastasis, but not with tumor size, depth of tumor invasion, differentiation, gender, or age. Overexpression of SIRT1 predicted a worse OS and higher TNM stage and lymphatic metastasis in Asian population especially in China. Therefore, overexpression of SIRT1 may promote lymphatic metastasis of cancers that lead to poor OS, DFS, EFS, and PFS. It is likely that SIRT1-mediated molecular events and biological processes could be an underlying mechanism for metastasis.

Our study is consistent with the most recent study by Wang et al. in that SIRT1 overexpression was significantly correlated with the OS in solid cancers, especially in liver cancer and lung cancer based on 7,369 cases from 37 studies and most of them are Asians (34). Consistently, the study by Hong et al showed that high SIRT1 expression correlated with vascular invasion and was not significantly correlated with overall survival rates in colon cancer (36). Study with 3024 patients by Wu et al showed that high SIRT1 expression predicts poor survival in non-colorectal gastrointestinal cancer, but not in colorectal cancer (35). SIRT1 expression was correlated with depth of invasion, lymph node metastasis and TNM stage and predicted a poor OS in colorectal cancer patients based on an analysis with seven studies (33). In an analysis of 1,650 patients in seven studies, high SIRT1 expression predicts a poor prognosis of gastric cancer patients and linked with patients' age, T stage, N stage, and tumor differentiation (32). Analysis by Cao et al. based on six studies involving 604 patients showed that SIRT1 expression was correlated with poor DFS and OS and high TNM stage and lymph node metastasis (31). However, we have performed study on survival and clinicopathological significance of SIRT1 expression in cancers more comprehensively. First, we included 63 eligible articles and a total of 13,138 participants in our study. These patients represented 9 countries and 16 cancer types as well as Asian and Caucasian ethnic groups. Second, we investigated both clinicopathological and prognostic significance of SIRT1 expression based on comprehensive clinical data and performed a series of subgroup analysis based on prognostic types, clinicopathological characteristics, cancer types, ethnic groups, countries. These stratifications provide more vehicles in understanding the survival and clinicopathological significance of SIRT1 expression in cancers.

There are also limitations in our study. Firstly, we found that heterogeneity existed in the meta-analysis as indicated by the *I*^2^ values. It is predictable because of presence of inter-study differences in study design (prospective and retrospective), enrolled populations, treatment regimen, duration of follow-up, outcome measures, and other study and clinical characteristics (127). The heterogeneity among the studies remained, despite the usage of a random-effects model and subgroup analyses (128). Secondly, there is publication bias for SIRT1 expression and prognosis or clinicopathological characteristics as indicated by asymmetry of funnel plots for OS, DFS, EFS, RFS, CCS, PFS, and clinicopathological characteristics. Thirdly, we barely explored the correlation between SIRT1 overexpression and patient survival in terms of clinical parameters. Other elements that may contribute to the heterogeneity, such as therapeutic regimen, pathological grade, body mass index, and mean age, were not analyzed due to the lack of sufficient data (129). Fourthly, we performed a quantitative meta-analysis based mostly on secondary data, which could lead to inaccurate results because of a shortage of original individual patient data (130). Finally, we conducted our study based on the mRNA expression of SIRT1 or the protein levels, although the changes in the mRNA and protein levels of SIRT1 are consistent in several cancer types (15, 16, 131, 132). The study by Hong et al who determined SIRT1 expression using immunohistochemistry showed similar results to ours study in relation with vascular invasion and overall survival rates in colon cancer (36). We should extensively investigate the prognostic and clinicopathological significance of SIRT1 expression at protein level in the future.

In conclusion, we have found that elevated expression of SIRT1 can predict poor OS, DFS, EFS, and PFS, but not with RFS or CCS, TNM stage, lymph node metastasis, and distant metastasis, but not tumor size, depth of tumor invasion, differentiation of cancers. Ethnic background has influence on the role of SIRT1 expression in predicting survival and clinicopathological characteristics of cancers. Overexpression of SIRT1 predicted a worse OS and higher TNM stage and lymphatic metastasis in Asian population especially in China. SIRT1-mediated molecular events and biological processes could be an underlying mechanism for metastasis and SIRT1 is a potential therapeutic target for inhibiting cancer metastasis. More studies that employ all prognostic indexes OS, DFS, EFS, RFS, CCS, and PFS or a full range of clinicopathological characteristics to study the role of SIRT1 expression in survival of patients with a specific cancer type, and mutations and polymorphisms of SIRT1 in cancers of different ethnic groups need to be further investigated in the future.

## Data Availability

All datasets generated for this study are included in the manuscript and the supplementary files.

## Author Contributions

MS, DH, XGu, and HZ participated in research design. MS, WZ, MD, SX, XGo, PL, HL, and JZ performed data analysis. MS and XGu wrote or contributed to the writing of the manuscript.

### Conflict of Interest Statement

The authors declare that the research was conducted in the absence of any commercial or financial relationships that could be construed as a potential conflict of interest.

## Supplementary Material

Supplementary Table 1 Databases searching terms. **(a)** Search criterion of Medline (via PubMed, from inception to April 1st, 2018) (*n* = 2397). **(b)** Search criterion of Embase (from 1966 to July 24, 2018) (*n* = 2460). **(c)** Search criterion of Cochrane Library (April 1st, 2018) (*n* = 20).

Supplementary Table 2 Results of quality assessment using the Newcastle–Ottawa Scale for the included studies.

Supplementary Table 3 Subgroup analysis for SIRT1 overexpression and the prognosis in cancers.

Supplementary Table 4 Subgroup analysis for SIRT1 overexpression and clinicopathological parameters in cancers.

Supplementary Table 5 Meta-regression analysis of heterogeneity for survival outcomes.

Supplementary Table 6 Meta-regression analysis of heterogeneity for clinicopathological parameters in cancers.

Supplementary Figure 1 Forest plot of subgroup analysis for SIRT1 overexpression and OS in cancers. **(A)** Cancer subgroup, **(B)** Country subgroup, **(C)** Ethnicity subgroup.

Supplementary Figure 2 Forest plot of subgroup analysis for SIRT1 overexpression and DFS in cancers. **(A)** Cancer subgroup, **(B)** Country subgroup, **(C)** Ethnicity subgroup.

Supplementary Figure 3 Forest plots of non-essential clinicopathological outcomes compared SIRT1 overexpression with underexpression. **(A)** Tumor size, **(B)** Depth of tumor invasion, **(C)** Differentiation, **(D)** Gender, **(E)** Age.

Supplementary Figure 4 Forest plot of subgroup analysis for SIRT1 overexpression and TNM stage in cancers. **(A)** Cancer subgroup, **(B)** Country subgroup, **(C)** Ethnicity subgroup.

Supplementary Figure 5 Forest plot of subgroup analysis for SIRT1 overexpression and distant metastasis in cancers. **(A)** Cancer subgroup, **(B)** Ethnicity subgroup.

Supplementary Figure 6 Forest plot of subgroup analysis for SIRT1 overexpression and lymphatic metastasis in cancers. **(A)** Country subgroup, **(B)** Ethnicity subgroup.

Supplementary Figure 7 Funnel plot for publication bias for SIRT1 expression and prognosis. **(A)** OS, **(B)** DFS, **(C)** EFS, **(D)** RFS, **(E)** CCS.

Supplementary Figure 8 Funnel plot for publication bias for SIRT1 expression and clinicopathological characteristics. **(A)** Age, **(B)** Gender, **(C)** Tumor stage, **(D)** Distant metastasis, **(E)** Lymphatic invasion, **(F)** Tumor size, **(G)** Depth of tumor invasion, **(H)** Differentiation.

## Abbreviations

OS: overall survival
SIRT1, silent information regulator 1: sirtuin-1
HR: hazard ratio
RR: relative risk
CI: confidence interval
DFS: disease free survival
EFS: event free survival
PFS: progress-free survival
RFS: recurrence-free survival
CCS: cancer-specific survival
NAD: nicotinamide adenine dinucleotide
q-PCR: quantitative real-time polymerase chain reaction
IHC: immunohistochemistry
ISH: *in situ* hybridization
NA: not available
NOS: newcastle-ottawa scale
NSCLC: non-small cell lung cancer
EOC: epithelial ovarian cancer
TNM, tumor, node: metastasis
MOOSE: meta-analysis of observational Studies in Epidemiology.
