# Supplementary material for: Survival and Clinicopathological Significance of SIRT1 Expression in Cancers: A Meta-Analysis
Source: Front Endocrinol (Lausanne). 2019 Mar 13;10:121. doi: 10.3389/fendo.2019.00121 (PMC6424908; doi:10.3389/fendo.2019.00121)
Supplement: Supplementary Table 1 — Databases searching terms. (a) Search criterion of Medline (via PubMed, from inception to April 1st, 2018) (n = 2397). (b) Search criterion of Embase (from 1966 to July 24, 2018) (n = 2460). (c) Search criterion of Cochrane Library (April 1st, 2018) (n = 20). [file Table_1.DOCX]

# Supplementary Table 1. Databases searching terms

Supplementary Table 1a. Search criterion of Medline (via PubMed, from inception to April 1st, 2018) (**n=2397**)

| Search  NO. | Query Results | Items found |
| --- | --- | --- |
| #1 | (((("sirtuin 1"[MeSH Terms] OR "sirtuin 1"[All Fields] OR "sirt1"[All Fields]) OR ("sirtuin 1"[MeSH Terms] OR "sirtuin 1"[All Fields])) OR SIR2[All Fields]) OR SIR2L1[All Fields]) OR SIR2alpha[All Fields] | 6823 |
| #2 | (((((("carcinoma"[MeSH Terms] OR "carcinoma"[All Fields]) OR (malig[tw] OR maliga[tw] OR maligaimedu[tw] OR maligan[tw] OR maliganacies[tw] OR maliganacy[tw] OR maliganancies[tw] OR maliganancy[tw] OR maliganant[tw] OR maliganat[tw] OR maligances[tw] OR maligancies[tw] OR maligancy[tw] OR maliganncies[tw] OR maligannt[tw] OR maligant[tw] OR maligeni[tw] OR maliger[tw] OR maligering[tw] OR malighi[tw] OR malighomas[tw] OR maligiphian[tw] OR maligita[tw] OR maliglancies[tw] OR maliglant[tw] OR maligmancies[tw] OR maligmancy[tw] OR maligmant[tw] OR maligment[tw] OR malign[tw] OR malign'[tw] OR maligna[tw] OR malignacies[tw] OR malignacy[tw] OR malignae[tw] OR malignamcy[tw] OR malignamt[tw] OR malignan[tw] OR malignana[tw] OR malignanacy[tw] OR malignanancy[tw] OR malignanant[tw] OR malignanat[tw] OR malignanc[tw] OR malignance[tw] OR malignances[tw] OR malignanci[tw] OR malignancics[tw] OR malignancie[tw] OR malignancie's[tw] OR malignancie`s[tw] OR malignancies[tw] OR malignancies'[tw] OR malignanciesin[tw] OR malignanciespublished[tw] OR malignanciestranslated[tw] OR malignancis[tw] OR malignanciy[tw] OR malignanct[tw] OR malignanctes[tw] OR malignancues[tw] OR malignancy[tw] OR malignancy'[tw] OR malignancy's[tw] OR malignancyand[tw] OR malignancyassociated[tw] OR malignancycase[tw] OR malignancycementation[tw] OR malignancyduring[tw] OR malignancyin[tw] OR malignancyinduced[tw] OR malignancyinvolvingthe[tw] OR malignancylike[tw] OR malignancyp[tw] OR malignancyr[tw] OR malignancys[tw] OR malignancysalivary[tw] OR malignancyseen[tw] OR malignancythat[tw] OR malignancyworldwide[tw] OR malignand[tw] OR malignang[tw] OR malignangies[tw] OR malignangt[tw] OR malignanices[tw] OR malignanicies[tw] OR malignanies[tw] OR malignano[tw] OR malignans[tw] OR malignanspluriorificial[tw] OR malignant[tw] OR malignant'[tw] OR malignant's[tw] OR malignantappearing[tw] OR malignantappendiceal[tw] OR malignantascitis[tw] OR malignantbiliary[tw] OR malignantbladder[tw] OR malignantcell[tw] OR malignante[tw] OR malignantfeature[tw] OR malignantfibrous[tw] OR malignantglioma[tw] OR malignantization[tw] OR malignantlesions[tw] OR malignantlike[tw] OR malignantly[tw] OR malignantlymphoma[tw] OR malignantmelanoma[tw] OR malignantneoplasm[tw] OR malignantneoplasms[tw] OR malignantnerve[tw] OR malignantneuroectodermal[tw] OR malignanton[tw] OR malignantperitoneal[tw] OR malignantrisk[tw] OR malignants[tw] OR malignantsyphilis[tw] OR malignanttransformation[tw] OR malignanttransforming[tw] OR malignanttumors[tw] OR malignanty[tw] OR malignanvies[tw] OR malignany[tw] OR malignas[tw] OR malignat[tw] OR malignated[tw] OR malignatization[tw] OR malignatized[tw] OR malignatn[tw] OR maligne[tw] OR maligned[tw] OR maligned'[tw] OR malignem[tw] OR malignement[tw] OR malignen[tw] OR malignencies[tw] OR malignency[tw] OR malignent[tw] OR maligner[tw] OR maligner'[tw] OR malignerers[tw] OR malignering[tw] OR malignes[tw] OR malignesation[tw] OR maligngroup[tw] OR maligni[tw] OR maligniances[tw] OR maligniant[tw] OR malignicies[tw] OR malignicity[tw] OR malignicy[tw] OR malignidad[tw] OR malignidade[tw] OR malignidades[tw] OR malignification[tw] OR malignified[tw] OR malignin[tw] OR maligning[tw] OR malignisans[tw] OR malignisation[tw] OR malignisations[tw] OR malignised[tw] OR malignismall[tw] OR malignit[tw] OR malignitas[tw] OR malignitasok[tw] OR malignitat[tw] OR malignitation[tw] OR malignitatsbestimmung[tw] OR malignitatsrate[tw] OR malignite[tw] OR malignitet[tw] OR malignities[tw] OR malignity[tw] OR malignium[tw] OR malignizacion[tw] OR malignizaiton[tw] OR malignization[tw] OR malignizations[tw] OR malignized[tw] OR malignlant[tw] OR malignment[tw] OR malignment'[tw] OR malignments[tw] OR malignn[tw] OR malignnancies[tw] OR malignnant[tw] OR malignnat[tw] OR maligno[tw] OR malignograms[tw] OR malignolipin[tw] OR malignom[tw] OR malignoma[tw] OR malignomas[tw] OR malignome[tw] OR malignomes[tw] OR malignoms[tw] OR malignomvorhersage[tw] OR malignos[tw] OR malignotoxic[tw] OR malignotoxicity[tw] OR malignotropic[tw] OR malignous[tw] OR maligns[tw] OR malignsancies[tw] OR malignt[tw] OR malignum[tw] OR malignum'[tw] OR malignus[tw] OR maligonomies[tw] OR maligrancies[tw] OR maligranda[tw] OR maliguant[tw] OR maliguda[tw] OR maligue[tw] OR maligum[tw] OR maligvant[tw])) OR (neoplas[tw] OR neoplasam[tw] OR neoplasams[tw] OR neoplascias[tw] OR neoplasctic[tw] OR neoplase[tw] OR neoplasi[tw] OR neoplasia[tw] OR neoplasia'[tw] OR neoplasia''[tw] OR neoplasia's[tw] OR neoplasia1[tw] OR neoplasia2[tw] OR neoplasiaa[tw] OR neoplasiae[tw] OR neoplasiafor[tw] OR neoplasiak[tw] OR neoplasialike[tw] OR neoplasialn[tw] OR neoplasias[tw] OR neoplasias'[tw] OR neoplasiaspecific[tw] OR neoplasiathe[tw] OR neoplasic[tw] OR neoplasica[tw] OR neoplasical[tw] OR neoplasicas[tw] OR neoplasico[tw] OR neoplasicos[tw] OR neoplasics[tw] OR neoplasie[tw] OR neoplasien[tw] OR neoplasies[tw] OR neoplasims[tw] OR neoplasing[tw] OR neoplasins[tw] OR neoplasiogenic[tw] OR neoplasique[tw] OR neoplasiques[tw] OR neoplasis[tw] OR neoplasitc[tw] OR neoplasitic[tw] OR neoplasle[tw] OR neoplaslic[tw] OR neoplaslms[tw] OR neoplasm[tw] OR neoplasm'[tw] OR neoplasm''[tw] OR neoplasm's[tw] OR neoplasm3[tw] OR neoplasma[tw] OR neoplasmal[tw] OR neoplasmama[tw] OR neoplasmand[tw] OR neoplasmas[tw] OR neoplasmata[tw] OR neoplasmatic[tw] OR neoplasmatica[tw] OR neoplasmaticae[tw] OR neoplasmatical[tw] OR neoplasmatically[tw] OR neoplasmaticus[tw] OR neoplasmatogenesis[tw] OR neoplasme[tw] OR neoplasmes[tw] OR neoplasmia[tw] OR neoplasmic[tw] OR neoplasmin[tw] OR neoplasmis[tw] OR neoplasmleft[tw] OR neoplasmn[tw] OR neoplasmns[tw] OR neoplasmogenesis[tw] OR neoplasmosis[tw] OR neoplasms[tw] OR neoplasms'[tw] OR neoplasms's[tw] OR neoplasmsis[tw] OR neoplasmsms[tw] OR neoplasmsoxaliplatin[tw] OR neoplasmsr[tw] OR neoplasmsv[tw] OR neoplasmswith[tw] OR neoplasmsworldwide[tw] OR neoplasmthe[tw] OR neoplasmtic[tw] OR neoplasmus[tw] OR neoplasmy[tw] OR neoplasoms[tw] OR neoplass[tw] OR neoplassia[tw] OR neoplassic[tw] OR neoplast[tw] OR neoplasta[tw] OR neoplastc[tw] OR neoplaster[tw] OR neoplastia[tw] OR neoplastic[tw] OR neoplastic'[tw] OR neoplastica[tw] OR neoplastical[tw] OR neoplastically[tw] OR neoplasticas[tw] OR neoplasticcells[tw] OR neoplastiche[tw] OR neoplastichuman[tw] OR neoplasticities[tw] OR neoplasticity[tw] OR neoplasticnom[tw] OR neoplastics[tw] OR neoplasticsyndromes[tw] OR neoplastictherapy[tw] OR neoplasticum[tw] OR neoplastie[tw] OR neoplasties[tw] OR neoplastiform[tw] OR neoplastiforme[tw] OR neoplastiforn[tw] OR neoplastigenesis[tw] OR neoplastigenic[tw] OR neoplastin[tw] OR neoplastine[tw] OR neoplastio[tw] OR neoplastioc[tw] OR neoplastische[tw] OR neoplastischer[tw] OR neoplastive[tw] OR neoplastmatic[tw] OR neoplastogenesis[tw] OR neoplastogenic[tw] OR neoplastogens[tw] OR neoplastoid[tw] OR neoplaston[tw] OR neoplasts[tw] OR neoplasty[tw] OR neoplasy[tw])) OR (oncol[tw] OR oncol'[tw] OR oncol1[tw] OR oncol13[tw] OR oncol26[tw] OR oncol7[tw] OR oncolab[tw] OR oncolaimus[tw] OR oncolaparoscopic[tw] OR oncolar[tw] OR oncolbx[tw] OR oncolead[tw] OR oncoleaking[tw] OR oncolectin[tw] OR oncolgeriatric[tw] OR oncolgical[tw] OR oncolgroup[tw] OR oncolgy[tw] OR oncolife[tw] OR oncoline[tw] OR oncolines[tw] OR oncolink[tw] OR oncolink'[tw] OR oncolink's[tw] OR oncolipid[tw] OR oncolipids[tw] OR oncolipids'[tw] OR oncolipin[tw] OR oncolipin's[tw] OR oncolitbank[tw] OR oncolites[tw] OR oncolitic[tw] OR oncollogically[tw] OR oncolnc[tw] OR oncolo[tw] OR oncolofical[tw] OR oncolofy[tw] OR oncolog[tw] OR oncolog'ia[tw] OR oncologi[tw] OR oncologia[tw] OR oncologia'[tw] OR oncologial[tw] OR oncologic[tw] OR oncologica[tw] OR oncological[tw] OR oncological'[tw] OR oncologicalandcosmetic[tw] OR oncologically[tw] OR oncologicals[tw] OR oncologicaly[tw] OR oncologicamente[tw] OR oncologicas[tw] OR oncologicchronic[tw] OR oncologiche[tw] OR oncologici[tw] OR oncologico[tw] OR oncologicos[tw] OR oncologics[tw] OR oncologictrade[tw] OR oncologicy[tw] OR oncologie[tw] OR oncologies[tw] OR oncologiests[tw] OR oncologik[tw] OR oncologiq[tw] OR oncologique[tw] OR oncologiques[tw] OR oncologisch[tw] OR oncologist[tw] OR oncologist'[tw] OR oncologist's[tw] OR oncologista[tw] OR oncologistpatient[tw] OR oncologists[tw] OR oncologists'[tw] OR oncologists'perceptions[tw] OR oncologists`knowledge[tw] OR oncologo[tw] OR oncologucal[tw] OR oncologue[tw] OR oncologues[tw] OR oncology[tw] OR oncology'[tw] OR oncology's[tw] OR oncology1[tw] OR oncologya[tw] OR oncologybasic[tw] OR oncologybest[tw] OR oncologyc[tw] OR oncologycal[tw] OR oncologycally[tw] OR oncologyclinical[tw] OR oncologygastrointestinal[tw] OR oncologyhas[tw] OR oncologylung[tw] OR oncologylymphoma[tw] OR oncologynutrition[tw] OR oncologyobjective[tw] OR oncologyprostate[tw] OR oncologysts[tw] OR oncologyt[tw] OR oncologyuunit[tw] OR oncologyzx[tw] OR oncoloic[tw] OR oncololgy[tw] OR oncolology[tw] OR oncoloqy[tw] OR oncolor[tw] OR oncolor's[tw] OR oncoloring[tw] OR oncolosits[tw] OR oncoloy[tw] OR oncoloyg[tw] OR oncolr[tw] OR oncoltyic[tw] OR oncolumn[tw] OR oncolyic[tw] OR oncolyitc[tw] OR oncolyn[tw] OR oncolyn's[tw] OR oncolys[tw] OR oncolysate[tw] OR oncolysates[tw] OR oncolyses[tw] OR oncolysin[tw] OR oncolysing[tw] OR oncolysis[tw] OR oncolytic[tw] OR oncolytic'[tw] OR oncolytical[tw] OR oncolytically[tw] OR oncolytics[tw] OR oncolyticum[tw] OR oncolyticvaccinia[tw] OR oncolyticvirotherapies[tw] OR oncolytoc[tw])) OR (tumor[tw] OR tumor'[tw] OR tumor''[tw] OR tumor''s[tw] OR tumor'growth[tw] OR tumor's[tw] OR tumor0[tw] OR tumor1[tw] OR tumor2[tw] OR tumor`s[tw] OR tumora[tw] OR tumora's[tw] OR tumorablation[tw] OR tumorablations[tw] OR tumorablative[tw] OR tumoracion[tw] OR tumoraciones[tw] OR tumoractivated[tw] OR tumoractivity[tw] OR tumoradapted[tw] OR tumoradc[tw] OR tumoradjacent[tw] OR tumorae[tw] OR tumoraffected[tw] OR tumoraffin[tw] OR tumoraffine[tw] OR tumoragenecity[tw] OR tumoragenic[tw] OR tumorahnliche[tw] OR tumorais[tw] OR tumoral[tw] OR tumoral'[tw] OR tumorale[tw] OR tumorales[tw] OR tumoralis[tw] OR tumorally[tw] OR tumoralp53[tw] OR tumoralpathology[tw] OR tumoralpotent[tw] OR tumorals[tw] OR tumoralstroma[tw] OR tumoralstromain[tw] OR tumorambulanz[tw] OR tumorand[tw] OR tumorangiogenesis[tw] OR tumorangiognesis[tw] OR tumorantigen[tw] OR tumorantigens[tw] OR tumorapa[tw] OR tumoraspect[tw] OR tumorassociated[tw] OR tumorassociated'[tw] OR tumoration[tw] OR tumorations[tw] OR tumoraux[tw] OR tumorbank[tw] OR tumorbasistherapie[tw] OR tumorbearers[tw] OR tumorbearing[tw] OR tumorbed[tw] OR tumorbedingten[tw] OR tumorbinding[tw] OR tumorbiogenesis[tw] OR tumorbiologic[tw] OR tumorbiological[tw] OR tumorbiologically[tw] OR tumorbiologie[tw] OR tumorbiology[tw] OR tumorbiopsy[tw] OR tumorblood[tw] OR tumorboard[tw] OR tumorboards[tw] OR tumorboder[tw] OR tumorboost[tw] OR tumorbox[tw] OR tumorbraf[tw] OR tumorbrain[tw] OR tumorbudding[tw] OR tumorby[tw] OR tumorcell[tw] OR tumorcellde[tw] OR tumorcelllines[tw] OR tumorcells[tw] OR tumorcellular[tw] OR tumorcenter[tw] OR tumorcenters[tw] OR tumorcerebri[tw] OR tumorcharged[tw] OR tumorchemosensitivity[tw] OR tumorchemotherapy[tw] OR tumorci[tw] OR tumorcidal[tw] OR tumorcide[tw] OR tumorclones[tw] OR tumorcode[tw] OR tumorcomponents[tw] OR tumorcontaining[tw] OR tumorcytogenetics[tw] OR tumorcytotoxic[tw] OR tumorcytotoxicity[tw] OR tumord[tw] OR tumordagger[tw] OR tumordebulking[tw] OR tumorderived[tw] OR tumordetektion[tw] OR tumordevelopment[tw] OR tumordforegoing[tw] OR tumordiagnostic[tw] OR tumordiathesis[tw] OR tumordicke[tw] OR tumordid[tw] OR tumordisease[tw] OR tumordissemination[tw] OR tumordokumentationssystem[tw] OR tumordosis[tw] OR tumordraining[tw] OR tumorduplicity[tw] OR tumore[tw] OR tumorearing[tw] OR tumorec[tw] OR tumorectomia[tw] OR tumorectomie[tw] OR tumorectomies[tw] OR tumorectomy[tw] OR tumored[tw] OR tumoregenecity[tw] OR tumoregenesis[tw] OR tumoregenic[tw] OR tumoregenicity[tw] OR tumoregensis[tw] OR tumoreginicity[tw] OR tumorellenes[tw] OR tumoremboli[tw] OR tumorembolie[tw] OR tumorembolisation[tw] OR tumoremia[tw] OR tumoren[tw] OR tumorendoprosteses[tw] OR tumorentfernung[tw] OR tumorentities[tw] OR tumorenucleation[tw] OR tumorep[tw] OR tumorepidemiologia[tw] OR tumorerkennung[tw] OR tumorerkrankungen[tw] OR tumores[tw] OR tumoret[tw] OR tumoretcomies[tw] OR tumoretomies[tw] OR tumorette[tw] OR tumorettes[tw] OR tumorevoked[tw] OR tumorex[tw] OR tumorexcision[tw] OR tumorexpressing[tw] OR tumorexstirpation[tw] OR tumorextension[tw] OR tumorextremely[tw] OR tumorexzisionen[tw] OR tumorforming[tw] OR tumorfree[tw] OR tumorfusions[tw] OR tumorgenecity[tw] OR tumorgenerasis[tw] OR tumorgenesis[tw] OR tumorgenic[tw] OR tumorgenicity[tw] OR tumorgeniesis[tw] OR tumorgenisis[tw] OR tumorgenom[tw] OR tumorgenome[tw] OR tumorgensis[tw] OR tumorgeschwulst[tw] OR tumorgewebe[tw] OR tumorglow[tw] OR tumorgrading[tw] OR tumorgraft[tw] OR tumorgraft'[tw] OR tumorgrafts[tw] OR tumorgrafts'[tw] OR tumorgrafttrade[tw] OR tumorgraphy[tw] OR tumorgroup[tw] OR tumorgrowth[tw] OR tumorh[tw] OR tumorhe[tw] OR tumorhead[tw] OR tumorhoming[tw] OR tumorhope[tw] OR tumorhost[tw] OR tumorhoz[tw] OR tumorhpd[tw] OR tumori[tw] OR tumoribus[tw] OR tumoric[tw] OR tumorical[tw] OR tumorici[tw] OR tumoricial[tw] OR tumoricid[tw] OR tumoricidal[tw] OR tumoricidal'[tw] OR tumoricidalactivity[tw] OR tumoricidaleffects[tw] OR tumoricidally[tw] OR tumoricidals[tw] OR tumoricide[tw] OR tumoricides[tw] OR tumoricidial[tw] OR tumoricidic[tw] OR tumoricity[tw] OR tumoridical[tw] OR tumoriferous[tw] OR tumoriform[tw] OR tumoriformis[tw] OR tumorifrons[tw] OR tumorigc[tw] OR tumorige[tw] OR tumorigeicity[tw] OR tumorigen[tw] OR tumorigenagent[tw] OR tumorigencicity[tw] OR tumorigencity[tw] OR tumorigency[tw] OR tumorigene[tw] OR tumorigenecities[tw] OR tumorigenecity[tw] OR tumorigeneic[tw] OR tumorigeneicity[tw] OR tumorigeneis[tw] OR tumorigeneisis[tw] OR tumorigeneity[tw] OR tumorigenenesis[tw] OR tumorigenenic[tw] OR tumorigenenicity[tw] OR tumorigenensis[tw] OR tumorigenes[tw] OR tumorigenese[tw] OR tumorigeneses[tw] OR tumorigenesis[tw] OR tumorigenesis'[tw] OR tumorigenesisassociated[tw] OR tumorigenesisby[tw] OR tumorigenesises[tw] OR tumorigenesisis[tw] OR tumorigenesisrelated[tw] OR tumorigenesiss[tw] OR tumorigenesity[tw] OR tumorigeness[tw] OR tumorigenetic[tw] OR tumorigenetically[tw] OR tumorigenety[tw] OR tumorigenic[tw] OR tumorigenic'[tw] OR tumorigenically[tw] OR tumorigenicities[tw] OR tumorigenicity[tw] OR tumorigenicity'[tw] OR tumorigenicity18[tw] OR tumorigenics[tw] OR tumorigenicty[tw] OR tumorigenicy[tw] OR tumorigenises[tw] OR tumorigenisis[tw] OR tumorigenisity[tw] OR tumorigenition[tw] OR tumorigenity[tw] OR tumorigenous[tw] OR tumorigens[tw] OR tumorigensis[tw] OR tumorigentic[tw] OR tumorigenticity[tw] OR tumorigenzcity[tw] OR tumorigesis[tw] OR tumoriginecity[tw] OR tumoriginesis[tw] OR tumoriginicity[tw] OR tumorignecity[tw] OR tumorignenesis[tw] OR tumorignesis[tw] OR tumorigram[tw] OR tumorilor[tw] OR tumorilysin[tw] OR tumorilysis[tw] OR tumorilytic[tw] OR tumorimeter[tw] OR tumorimmunolocalization[tw] OR tumorimmunologische[tw] OR tumorimmunologists[tw] OR tumorimtis[tw] OR tumorimvasion[tw] OR tumorin[tw] OR tumorindependent[tw] OR tumorinduced[tw] OR tumorinducing[tw] OR tumorinduction[tw] OR tumorinfiltrated[tw] OR tumorinfiltrating[tw] OR tumorinfiltration[tw] OR tumoring[tw] OR tumoringenic[tw] OR tumoringenicity[tw] OR tumorinhibiting[tw] OR tumorinhibitor[tw] OR tumorinhibitory[tw] OR tumorinitiating[tw] OR tumorinitiation[tw] OR tumorinogenesis[tw] OR tumorinogens[tw] OR tumorinvasion[tw] OR tumoriods[tw] OR tumoriogenesis[tw] OR tumoriogenic[tw] OR tumorionline[tw] OR tumoris[tw] OR tumorisation[tw] OR tumorisiphum[tw] OR tumorisphere[tw] OR tumorispheres[tw] OR tumoristasis[tw] OR tumoristatic[tw] OR tumoristic[tw] OR tumoritoxic[tw] OR tumoritoxicity[tw] OR tumoritrophism[tw] OR tumoritropic[tw] OR tumoritropism[tw] OR tumorivascular[tw] OR tumorization[tw] OR tumorized[tw] OR tumorjellemzok[tw] OR tumork[tw] OR tumorkaryotype[tw] OR tumorkines[tw] OR tumorkranke[tw] OR tumorkranken[tw] OR tumorl[tw] OR tumorleri[tw] OR tumorless[tw] OR tumorlet[tw] OR tumorlet'[tw] OR tumorlet's[tw] OR tumorlets[tw] OR tumorlia[tw] OR tumorlike[tw] OR tumorload[tw] OR tumorlocalization[tw] OR tumorlocalizer[tw] OR tumorlocalizing[tw] OR tumorlocation[tw] OR tumorlocationwere[tw] OR tumorlymph[tw] OR tumorlysate[tw] OR tumorlyse[tw] OR tumorlysis[tw] OR tumorlytic[tw] OR tumorlyzing[tw] OR tumormakers[tw] OR tumormalignancy[tw] OR tumormap[tw] OR tumormarker[tw] OR tumormarkerresponse[tw] OR tumormarkers[tw] OR tumormass[tw] OR tumormedulloblastoma[tw] OR tumormetastasen[tw] OR tumormetastasis[tw] OR tumormetric[tw] OR tumormetrical[tw] OR tumormetry[tw] OR tumormfurthermore[tw] OR tumormicroenvironment[tw] OR tumormicrovironment[tw] OR tumormitosen[tw] OR tumorml[tw] OR tumormodels[tw] OR tumormrglitt[tw] OR tumormsc[tw] OR tumornachweis[tw] OR tumornecrosis[tw] OR tumornecrosisfactor[tw] OR tumornecross[tw] OR tumornecrotic[tw] OR tumornekrosefaktor[tw] OR tumorneoplasia[tw] OR tumornephrectomy[tw] OR tumornext[tw] OR tumornode[tw] OR tumornodemetastasis[tw] OR tumornodes[tw] OR tumornodesmetastasis[tw] OR tumornodules[tw] OR tumoro[tw] OR tumorobiological[tw] OR tumorocentric[tw] OR tumorocidal[tw] OR tumorogenecity[tw] OR tumorogeneity[tw] OR tumorogenesis[tw] OR tumorogenesity[tw] OR tumorogenetic[tw] OR tumorogenic[tw] OR tumorogenical[tw] OR tumorogenicity[tw] OR tumorogenisity[tw] OR tumorogenity[tw] OR tumorogensis[tw] OR tumorography[tw] OR tumoroid[tw] OR tumoroidal[tw] OR tumoroidea[tw] OR tumoroids[tw] OR tumorok[tw] OR tumorolytic[tw] OR tumoromicronus[tw] OR tumoronecroticance[tw] OR tumorons[tw] OR tumoropathic[tw] OR tumoropathy[tw] OR tumoroperationen[tw] OR tumorophilic[tw] OR tumoropsy[tw] OR tumororchiectomy[tw] OR tumororetinal[tw] OR tumoros[tw] OR tumoroshperes[tw] OR tumorosphere[tw] OR tumorospheres[tw] OR tumorosphore[tw] OR tumorossejt[tw] OR tumorostatic[tw] OR tumorosteolysis[tw] OR tumorosus[tw] OR tumorotrophic[tw] OR tumorotropic[tw] OR tumorous[tw] OR tumorous'[tw] OR tumorously[tw] OR tumorousone[tw] OR tumorp[tw] OR tumorparameters[tw] OR tumorpathogenesis[tw] OR tumorpatienten[tw] OR tumorpatients[tw] OR tumorpenetrating[tw] OR tumorpharmacology[tw] OR tumorpil[tw] OR tumorplatelet[tw] OR tumorplextrade[tw] OR tumorplos[tw] OR tumorpositive[tw] OR tumorpreventive[tw] OR tumorprism3d[tw] OR tumorprogression[tw] OR tumorpromoter[tw] OR tumorpromoting[tw] OR tumorpromotion[tw] OR tumorpromotor[tw] OR tumorprone[tw] OR tumorprotective[tw] OR tumorprotein[tw] OR tumorr[tw] OR tumorradicality[tw] OR tumorral[tw] OR tumorreactive[tw] OR tumorrecidiva[tw] OR tumorrecurrence[tw] OR tumorreducing[tw] OR tumorreduction[tw] OR tumorregionen[tw] OR tumorregression[tw] OR tumorregressive[tw] OR tumorrelated[tw] OR tumorreleased[tw] OR tumorreport[tw] OR tumorresection[tw] OR tumorresektion[tw] OR tumorresiduals[tw] OR tumorresiduen[tw] OR tumorresistance[tw] OR tumorresponse[tw] OR tumorresponsewas[tw] OR tumorrest[tw] OR tumorrests[tw] OR tumors[tw] OR tumors'[tw] OR tumors's[tw] OR tumors1[tw] OR tumorsa[tw] OR tumorsand[tw] OR tumorsat[tw] OR tumorscan[tw] OR tumorscanning[tw] OR tumorscape[tw] OR tumorschest[tw] OR tumorschmerzen[tw] OR tumorschnitt[tw] OR tumorscintigraphy[tw] OR tumorsclinical[tw] OR tumorscreening[tw] OR tumorsdatabase[tw] OR tumorsections[tw] OR tumorsejtvonalak[tw] OR tumorselective[tw] OR tumorsera[tw] OR tumorsgroup[tw] OR tumorshad[tw] OR tumorshas[tw] OR tumorshowed[tw] OR tumorsi[tw] OR tumorsimulating[tw] OR tumorsin[tw] OR tumorsis[tw] OR tumorsite[tw] OR tumorsize[tw] OR tumorsliver[tw] OR tumorsmcid[tw] OR tumorsn[tw] OR tumorsof[tw] OR tumorsor[tw] OR tumorspace[tw] OR tumorspecific[tw] OR tumorsphere[tw] OR tumorsphereforming[tw] OR tumorspheres[tw] OR tumorspheric[tw] OR tumorspread[tw] OR tumorsquareafter[tw] OR tumorsranged[tw] OR tumorss[tw] OR tumorsstrategies[tw] OR tumorstadium[tw] OR tumorstage[tw] OR tumorstages[tw] OR tumorstaging[tw] OR tumorstatic[tw] OR tumorstatus[tw] OR tumorstemcell[tw] OR tumorsthat[tw] OR tumorsthatare[tw] OR tumorstroma[tw] OR tumorstromal[tw] OR tumorsupplying[tw] OR tumorsuppressing[tw] OR tumorsuppression[tw] OR tumorsuppressive[tw] OR tumorsuppressor[tw] OR tumorsuppressors[tw] OR tumorsupressor[tw] OR tumorsurgery[tw] OR tumorsurrounding[tw] OR tumorsuspected[tw] OR tumorswas[tw] OR tumorswere[tw] OR tumorswith[tw] OR tumorsyielded[tw] OR tumorsyndrom[tw] OR tumorsyndrome[tw] OR tumorszindromak[tw] OR tumorszuppresszor[tw] OR tumortargeted[tw] OR tumortargeting[tw] OR tumortherapie[tw] OR tumortherapie'[tw] OR tumortherapies[tw] OR tumortherapy[tw] OR tumortissue[tw] OR tumortissues[tw] OR tumorto[tw] OR tumortoorgan[tw] OR tumortoxic[tw] OR tumortoxicity[tw] OR tumortr[tw] OR tumortracer[tw] OR tumortragenden[tw] OR tumortrophic[tw] OR tumortropic[tw] OR tumortropism[tw] OR tumorts[tw] OR tumortumor[tw] OR tumortumour[tw] OR tumortx[tw] OR tumortype[tw] OR tumortypes[tw] OR tumoru[tw] OR tumoruigenesis[tw] OR tumorum[tw] OR tumoruous[tw] OR tumorurs[tw] OR tumorus[tw] OR tumorvirgae[tw] OR tumorvirus[tw] OR tumorviruses[tw] OR tumorvolumen[tw] OR tumorvolumes[tw] OR tumorvolumetrie[tw] OR tumorvorsorge[tw] OR tumorwachstum[tw] OR tumorwas[tw] OR tumorwasmobilized[tw] OR tumorweight[tw])) OR ("neoplasms"[MeSH Terms] OR "neoplasms"[All Fields]) OR (adenocarcinom[tw] OR adenocarcinoma[tw] OR adenocarcinoma'[tw] OR adenocarcinoma's[tw] OR adenocarcinomaa[tw] OR adenocarcinomaand[tw] OR adenocarcinomaassociated[tw] OR adenocarcinomacancer[tw] OR adenocarcinomacell[tw] OR adenocarcinomacells[tw] OR adenocarcinomacortactin[tw] OR adenocarcinomadagger[tw] OR adenocarcinomademonstrated[tw] OR adenocarcinomaderived[tw] OR adenocarcinomadetection[tw] OR adenocarcinomagenesis[tw] OR adenocarcinomahave[tw] OR adenocarcinomain[tw] OR adenocarcinomal[tw] OR adenocarcinomalike[tw] OR adenocarcinomaof[tw] OR adenocarcinomapatients[tw] OR adenocarcinomaprostatic[tw] OR adenocarcinomas[tw] OR adenocarcinomas'[tw] OR adenocarcinomaswith[tw] OR adenocarcinomat2an2m0[tw] OR adenocarcinomata[tw] OR adenocarcinomatas[tw] OR adenocarcinomateuse[tw] OR adenocarcinomathan[tw] OR adenocarcinomatic[tw] OR adenocarcinomatosis[tw] OR adenocarcinomatous[tw] OR adenocarcinomawas[tw] OR adenocarcinomawho[tw] OR adenocarcinome[tw] OR adenocarcinomes[tw] OR adenocarcinomia[tw] OR adenocarcinomic[tw] OR adenocarcinomist[tw] OR adenocarcinomists[tw] OR adenocarcinomma[tw] OR adenocarcinomna[tw] OR adenocarcinomnas[tw] OR adenocarcinomous[tw] OR adenocarcinoms[tw] OR adenocarcinomu[tw]) AND (cancer[tw] OR cancer'[tw] OR cancer''[tw] OR cancer'and[tw] OR cancer's[tw] OR cancer'screening'[tw] OR cancer1[tw] OR cancer1,2[tw] OR cancer10[tw] OR cancer14[tw] OR cancer2[tw] OR cancer2011[tw] OR cancer2015[tw] OR cancer29a[tw] OR cancer3d[tw] OR cancer4[tw] OR cancer411[tw] OR cancer5[tw] OR cancer6[tw] OR cancer68ga[tw] OR cancera[tw] OR canceraccomplishments[tw] OR canceractivity[tw] OR canceradjacent[tw] OR cancerae[tw] OR canceran[tw] OR cancerand[tw] OR cancerandmetabolism[tw] OR cancerangiogenesis[tw] OR cancerantigens[tw] OR cancerappears[tw] OR cancerare[tw] OR cancerarray[tw] OR cancerassociate[tw] OR cancerassociated[tw] OR cancerat[tw] OR cancerate[tw] OR cancerated[tw] OR canceration[tw] OR cancerations[tw] OR canceraudit[tw] OR canceraustralia[tw] OR cancerawareness[tw] OR cancerb[tw] OR cancerbackup[tw] OR cancerbacup[tw] OR cancerbacup's[tw] OR cancerbase[tw] OR cancerbearing[tw] OR cancerbio[tw] OR cancerbioassays[tw] OR cancerbiobank[tw] OR cancerbioinformatics[tw] OR cancerbiomarkers[tw] OR cancerbiostats[tw] OR cancerbridges[tw] OR cancerbush[tw] OR cancercare[tw] OR cancercarelinks[tw] OR cancercareontario[tw] OR cancercases[tw] OR cancercd[tw] OR cancercell[tw] OR cancercelllineclassificationsampleimages[tw] OR cancercells[tw] OR cancercenter[tw] OR cancercentrum[tw] OR cancerchatcanada[tw] OR cancerchemoprevention[tw] OR cancerchemotherapy[tw] OR cancercidal[tw] OR cancercinogenesis[tw] OR cancerclinical[tw] OR cancercolorectal[tw] OR cancercompared[tw] OR cancerconsultants[tw] OR cancercontaining[tw] OR cancercontribution[tw] OR cancercontrol[tw] OR cancercope[tw] OR cancercostmod[tw] OR cancercrc[tw] OR cancercsp[tw] OR cancercyto[tw] OR cancercytochrome[tw] OR cancerd2[tw] OR cancerdagger[tw] OR cancerdata[tw] OR cancerdb[tw] OR cancerderived[tw] OR cancerdetecting[tw] OR cancerdetection[tw] OR cancerdetector[tw] OR cancerdetector'[tw] OR cancerdevelopment[tw] OR cancerdiscover[tw] OR cancerdp[tw] OR cancerdr[tw] OR cancerdriving[tw] OR cancerdrugs[tw] OR cancerduring[tw] OR cancerdusein[tw] OR cancere[tw] OR cancered[tw] OR cancerembryonic[tw] OR canceremia[tw] OR cancerendocrine[tw] OR cancereous[tw] OR cancerequol[tw] OR canceres[tw] OR cancerescreening[tw] OR cancerest[tw] OR cancereuse[tw] OR cancereuses[tw] OR cancereux[tw] OR cancerexosome[tw] OR cancerf[tw] OR cancerfit[tw] OR cancerflat[tw] OR cancerfonden[tw] OR cancerfor[tw] OR cancerfree[tw] OR cancerfusiongenes[tw] OR cancergamadb[tw] OR cancergazing[tw] OR cancergd[tw] OR cancergen[tw] OR cancergene[tw] OR cancergenes[tw] OR cancergenetics[tw] OR cancergeneticsstorybank[tw] OR cancergenic[tw] OR cancergenome[tw] OR cancergenomeinterpreter[tw] OR cancergenomics[tw] OR cancergenomicscloud[tw] OR cancergogenic[tw] OR cancergram[tw] OR cancergrams[tw] OR cancergraph[tw] OR cancergrid[tw] OR cancerguides[tw] OR cancerguidesa[tw] OR cancerhad[tw] OR cancerhas[tw] OR cancerhazard[tw] OR cancerhbv[tw] OR cancerhelp[tw] OR cancerhelpuk[tw] OR cancerhemihypoperfusion[tw] OR cancerhep[tw] OR cancerhsp[tw] OR canceri[tw] OR canceri's[tw] OR canceric[tw] OR cancericidal[tw] OR cancericidals[tw] OR canceridal[tw] OR cancerides[tw] OR cancerification[tw] OR cancerigen[tw] OR cancerigenesis[tw] OR cancerigenic[tw] OR cancerigenically[tw] OR cancerigenous[tw] OR cancerigens[tw] OR cancerii[tw] OR cancerilla[tw] OR cancerimages[tw] OR cancerimagingarchive[tw] OR cancerimmunity[tw] OR cancerimmunotherapy[tw] OR cancerin[tw] OR cancerina[tw] OR cancerina'[tw] OR cancerincidence[tw] OR cancerincidences[tw] OR cancerinduced[tw] OR cancerinformatics[tw] OR cancerinitiating[tw] OR cancerinogenic[tw] OR cancerinpregnancy[tw] OR cancerinstitute[tw] OR canceriological[tw] OR cancerious[tw] OR canceris[tw] OR cancerisable[tw] OR cancerisation[tw] OR cancerisation'[tw] OR cancerised[tw] OR cancerit[tw] OR cancerizable[tw] OR cancerizacion[tw] OR cancerization[tw] OR cancerization'[tw] OR cancerizations[tw] OR cancerize[tw] OR cancerized[tw] OR canceriziation[tw] OR cancerk[tw] OR cancerkin[tw] OR cancerl[tw] OR cancerlandscapes[tw] OR cancerlectin[tw] OR cancerlectindb[tw] OR cancerlectins[tw] OR cancerless[tw] OR cancerlike[tw] OR cancerline[tw] OR cancerlink[tw] OR cancerlinks[tw] OR cancerlinq[tw] OR cancerlinq's[tw] OR cancerlit[tw] OR cancerlite[tw] OR cancerlocator[tw] OR cancerma[tw] OR cancermap[tw] OR cancermarkers[tw] OR cancermath[tw] OR cancermda[tw] OR cancermediated[tw] OR cancermetabolite[tw] OR cancermia[tw] OR cancerminer[tw] OR cancermodels[tw] OR cancermondial[tw] OR cancermutationanalysis[tw] OR cancern[tw] OR cancernation[tw] OR cancernegative[tw] OR cancerness[tw] OR cancerness'[tw] OR cancernet[tw] OR cancernext[tw] OR cancerni[tw] OR cancernichibo[tw] OR cancernogenesis[tw] OR cancernomograms[tw] OR cancernursing[tw] OR cancero[tw] OR canceroblastic[tw] OR cancerocerebral[tw] OR cancerocid[tw] OR cancerocidal[tw] OR canceroderms[tw] OR canceroenic[tw] OR cancerof[tw] OR cancerogen[tw] OR cancerogena[tw] OR cancerogene[tw] OR cancerogenenic[tw] OR cancerogenensis[tw] OR cancerogeneous[tw] OR cancerogenes[tw] OR cancerogenese[tw] OR cancerogenesi[tw] OR cancerogenesis[tw] OR cancerogenetic[tw] OR cancerogeni[tw] OR cancerogenic[tw] OR cancerogenicity[tw] OR cancerogenics[tw] OR cancerogenisation[tw] OR cancerogenity[tw] OR cancerogeniv[tw] OR cancerogenous[tw] OR cancerogens[tw] OR cancerogensis[tw] OR cancerogenus[tw] OR cancerola[tw] OR cancerolog'ia[tw] OR cancerologia[tw] OR cancerologic[tw] OR cancerological[tw] OR cancerologically[tw] OR cancerologie[tw] OR cancerologie'[tw] OR cancerologiedes[tw] OR cancerologist[tw] OR cancerologists[tw] OR cancerologues[tw] OR cancerology[tw] OR cancerolysin[tw] OR cancerolysis[tw] OR cancerolytic[tw] OR canceromatosis[tw] OR canceromatous[tw] OR cancerome[tw] OR cancerometastasis[tw] OR cancerometry[tw] OR canceromics[tw] OR canceromimetic[tw] OR canceronly[tw] OR cancerophilia[tw] OR cancerophilic[tw] OR cancerophobia[tw] OR cancerophobic[tw] OR canceropole[tw] OR canceropoles[tw] OR canceroprevention[tw] OR canceroprotective[tw] OR cancerordouble[tw] OR cancerosa[tw] OR canceroselectivity[tw] OR canceroses[tw] OR cancerosis[tw] OR cancerosis'[tw] OR cancerosity[tw] OR cancerostasis[tw] OR cancerostat[tw] OR cancerostatic[tw] OR cancerostatica[tw] OR cancerostatical[tw] OR cancerostatically[tw] OR cancerostaticas[tw] OR cancerostatics[tw] OR cancerostatis[tw] OR cancerotest[tw] OR cancerotherapy[tw] OR cancerotoxic[tw] OR cancerours[tw] OR cancerous[tw] OR cancerous'[tw] OR cancerously[tw] OR cancerouslymphoblasts[tw] OR cancerousous[tw] OR cancerouspdomains[tw] OR canceroverall[tw] OR canceroverly[tw] OR canceroverview[tw] OR cancerpaedia[tw] OR cancerpaedia'[tw] OR cancerpage[tw] OR cancerpain[tw] OR cancerpatients[tw] OR cancerpdf[tw] OR cancerpertinent[tw] OR cancerpetregistry[tw] OR cancerphobes[tw] OR cancerphobia[tw] OR cancerphobia'[tw] OR cancerphobic[tw] OR cancerphobics[tw] OR cancerphytochemical[tw] OR cancerplan[tw] OR cancerplex[tw] OR cancerpositive[tw] OR cancerppd[tw] OR cancerpppm[tw] OR cancerprecancer[tw] OR cancerpredisposing[tw] OR cancerpredisposition[tw] OR cancerpreventionscotland[tw] OR cancerpreventive[tw] OR cancerprogress[tw] OR cancerpromoting[tw] OR cancerprone[tw] OR cancerprophylaxis[tw] OR cancerprotective[tw] OR cancerproteomics[tw] OR cancerproview[tw] OR cancerr[tw] OR cancerradical[tw] OR cancerradioiodine[tw] OR cancerrealism[tw] OR cancerregional[tw] OR cancerregisterist[tw] OR cancerregistration[tw] OR cancerregistry[tw] OR cancerrelated[tw] OR cancerremains[tw] OR cancerreport[tw] OR cancerres[tw] OR cancerresearch[tw] OR cancerresearchuk[tw] OR cancerresource[tw] OR cancerresources[tw] OR cancerrisk[tw] OR cancerriskbiomarkers[tw] OR cancerrriskmanagement[tw] OR cancerrxgene[tw] OR cancers[tw] OR cancers'[tw] OR cancersadmitted[tw] OR cancersamples[tw] OR cancersassociated[tw] OR cancerscan[tw] OR cancerscantrade[tw] OR cancerscells[tw] OR cancerscope[tw] OR cancerscreening[tw] OR cancersea[tw] OR cancerseek[tw] OR cancersim[tw] OR cancersin[tw] OR cancersnon[tw] OR cancersoral[tw] OR cancersourcern[tw] OR cancerspace[tw] OR cancerspecific[tw] OR cancersplicingqtl[tw] OR cancersselected[tw] OR cancerssubmitted[tw] OR cancerstatic[tw] OR cancerstats[tw] OR cancersthan[tw] OR cancerstroma[tw] OR cancersubtypes[tw] OR cancersupportsource[tw] OR cancersurvivorlink[tw] OR cancersurvivors[tw] OR cancerswas[tw] OR cancerswere[tw] OR cancersysdb[tw] OR cancert[tw] OR cancertargeted[tw] OR cancertargeting[tw] OR cancertelsys[tw] OR cancertestis[tw] OR cancerthe[tw] OR cancertherapeutic[tw] OR cancertherapy[tw] OR cancerthis[tw] OR cancertiming[tw] OR cancertissue[tw] OR cancertool[tw] OR cancertope[tw] OR cancertrade[tw] OR cancertreated[tw] OR cancertreatment[tw] OR cancertrials[tw] OR cancertumorigenesis[tw] OR cancertwin[tw] OR cancertype[tw] OR cancertypeid[tw] OR cancertypes[tw] OR canceru[tw] OR cancerued[tw] OR cancerusing[tw] OR cancervax[tw] OR cancervia[tw] OR cancerview[tw] OR cancervih[tw] OR cancerving[tw] OR cancervirus[tw] OR cancervive[tw] OR cancerwas[tw] OR cancerwe[tw] OR cancerweb[tw] OR cancerwer[tw] OR cancerwere[tw] OR cancerwith[tw] OR cancerwithout[tw] OR cancerworking[tw] OR cancerworld[tw] OR cancerwort[tw] OR cancerxenografts[tw] OR canceryear[tw] OR cancerzap[tw] OR cancerzation[tw])) OR ("tumour"[All Fields] OR "neoplasms"[MeSH Terms] OR "neoplasms"[All Fields] OR "tumor"[All Fields]) OR ("neoplasms"[MeSH Terms] OR "neoplasms"[All Fields] OR "cancer"[All Fields]) OR (sarcom[tw] OR sarcoma[tw] OR sarcoma'[tw] OR sarcoma''[tw] OR sarcoma's[tw] OR sarcoma180[tw] OR sarcomaa[tw] OR sarcomaassociated[tw] OR sarcomabearing[tw] OR sarcomabotryoidal[tw] OR sarcomae[tw] OR sarcomagen[tw] OR sarcomagenesis[tw] OR sarcomagenesis'[tw] OR sarcomagenetic[tw] OR sarcomagenic[tw] OR sarcomagens[tw] OR sarcomaia[tw] OR sarcomaidcs[tw] OR sarcomaina[tw] OR sarcomal[tw] OR sarcomaleukemia[tw] OR sarcomaleukosis[tw] OR sarcomalike[tw] OR sarcomania[tw] OR sarcomaof[tw] OR sarcomaproducing[tw] OR sarcomarosis[tw] OR sarcomartu[tw] OR sarcomas[tw] OR sarcomas'[tw] OR sarcomastgophora[tw] OR sarcomastigophora[tw] OR sarcomastigophoran[tw] OR sarcomastigota[tw] OR sarcomat[tw] OR sarcomata[tw] OR sarcomata's[tw] OR sarcomataous[tw] OR sarcomateuse[tw] OR sarcomatic[tw] OR sarcomatid[tw] OR sarcomatiod[tw] OR sarcomatization[tw] OR sarcomatodes[tw] OR sarcomatogen[tw] OR sarcomatogenesis[tw] OR sarcomatogenic[tw] OR sarcomatogenous[tw] OR sarcomatoid[tw] OR sarcomatoid'[tw] OR sarcomatoide[tw] OR sarcomatoides[tw] OR sarcomatome[tw] OR sarcomatosa[tw] OR sarcomatose[tw] OR sarcomatoses[tw] OR sarcomatosia[tw] OR sarcomatosis[tw] OR sarcomatosum[tw] OR sarcomatouos[tw] OR sarcomatous[tw] OR sarcomatous'[tw] OR sarcomatouse[tw] OR sarcomatouslike[tw] OR sarcomatously[tw] OR sarcomatransformed[tw] OR sarcomatus[tw] OR sarcomavirus[tw] OR sarcome[tw] OR sarcomedium[tw] OR sarcomeginal[tw] OR sarcomejine[tw] OR sarcomelanin[tw] OR sarcomelelicope[tw] OR sarcomelicope[tw] OR sarcomelicopespecies[tw] OR sarcomenia[tw] OR sarcomer[tw] OR sarcomera[tw] OR sarcomeras[tw] OR sarcomercic[tw] OR sarcomere[tw] OR sarcomere'[tw] OR sarcomere's[tw] OR sarcomeregenesis[tw] OR sarcomeres[tw] OR sarcomeres'[tw] OR sarcomeric[tw] OR sarcomerical[tw] OR sarcomerically[tw] OR sarcomericas[tw] OR sarcomericlocalization[tw] OR sarcomericos[tw] OR sarcomerization[tw] OR sarcomero[tw] OR sarcomerogenesis[tw] OR sarcomerogensis[tw] OR sarcomeropathy[tw] OR sarcomeroptosis[tw] OR sarcomeros[tw] OR sarcomers[tw] OR sarcomes[tw] OR sarcomesothelioma[tw] OR sarcometic[tw] OR sarcometin[tw] OR sarcometric[tw] OR sarcomi[tw] OR sarcomic[tw] OR sarcomililatins[tw] OR sarcomililatol[tw] OR sarcomla[tw] OR sarcomma[tw] OR sarcomna[tw] OR sarcomnere[tw] OR sarcomogeneous[tw] OR sarcomogenesis[tw] OR sarcomogenic[tw] OR sarcomogenous[tw] OR sarcomonad[tw] OR sarcomonadea[tw] OR sarcomonads[tw] OR sarcomotoid[tw] OR sarcomplamsic[tw] OR sarcomplasmic[tw] OR sarcoms[tw] OR sarcomsome[tw] OR sarcomucosis[tw] OR sarcomy[tw] OR sarcomyces[tw] OR sarcomycin[tw] OR sarcomyogenesis[tw] OR sarcomyoma[tw] OR sarcomyopathies[tw] OR sarcomyopathy[tw]) | 4194418 |
| #3 | #1 AND #2 | 2397 |

##### Supplementary Table 1b. Search criterion of Embase (from 1966 to July 24, 2018) (n=2460)

| Search  NO. | Query Results | Items found |
| --- | --- | --- |
| #20 | #12 AND #19 | 2460 |
| #19 | #13 OR #14 OR #15 OR #16 OR #17 OR #18 | 9961 |
| #18 | 'sirtuin 1':ab,ti | 2229 |
| #17 | 'silent mating type information regulation 2 homolog-1' | 139 |
| #16 | sir2alpha:ab,ti | 3 |
| #15 | sir2l1:ab,ti | 1 |
| #14 | sir2:ab,ti | 1299 |
| #13 | 'sirt1' | 8472 |
| #12 | #1 OR #2 OR #3 OR #4 OR #5 OR #6 OR #7 OR #8 OR #9 OR #10 OR #11 | 5355730 |
| #11 | sarcom*:ab,ti | 134593 |
| #10 | cancer:ab,ti | 2073322 |
| #9 | tumour:ab,ti | 297527 |
| #8 | cancer*:ab,ti | 2168230 |
| #7 | adenocarcinom*:ab,ti | 192452 |
| #6 | 'neoplasms'/exp | 4566831 |
| #5 | tumor*:ab,ti | 1753850 |
| #4 | oncol*:ab,ti | 237021 |
| #3 | neoplas*:ab,ti | 339134 |
| #2 | malig*:ab,ti | 759633 |
| #1 | 'carcinoma'/exp | 1163326 |

##### Supplementary Table 1c. Search criterion of Cochrane Library (April 1st, 2018) (n=20)

| Search  NO. | Query Results | Items found |
| --- | --- | --- |
| #1 | (sirtuin 1):ti,ab,kw OR (SIRT1):ti,ab,kw OR (SIR2):ti,ab,kw OR (SIR2L1):ti,ab,kw OR (SIR2alpha):ti,ab,kw | 148 |
| #2 | MeSH descriptor: [Carcinoma] explode all trees | 11658 |
| #3 | (malig*):ti,ab,kw OR (neoplas*):ti,ab,kw OR (oncol*):ti,ab,kw OR (tumor*):ti,ab,kw OR (adenocarcinom*):ti,ab,kw | 113491 |
| #4 | (adenocarcinom*):ti,ab,kw | 6819 |
| #5 | (cancer*):ti,ab,kw | 110638 |
| #6 | (tumour):ti,ab,kw | 52316 |
| #7 | (cancer):ti,ab,kw | 110592 |
| #8 | (sarcom*):ti,ab,kw | 2000 |
| #9 | #2 OR #3 OR #4 OR #5 OR #6 OR #7 OR #8 | 154112 |
| #10 | #1 AND #9 | 20 |
