# Supplementary material for: Survival and Clinicopathological Significance of SIRT1 Expression in Cancers: A Meta-Analysis
Source: Front Endocrinol (Lausanne). 2019 Mar 13;10:121. doi: 10.3389/fendo.2019.00121 (PMC6424908; doi:10.3389/fendo.2019.00121)
Supplement: Supplementary Table 2 — Results of quality assessment using the Newcastle–Ottawa Scale for the included studies. [file Table_2.DOCX]

**Supplementary Table 2.** Results of quality assessment using the Newcastle–Ottawa Scale for the included studies

| No. | Study | Selection |  |  |  | Comparability | Exposure |  |  | Scores |
| --- | --- | --- | --- | --- | --- | --- | --- | --- | --- | --- |
|  |  | Is the case definition adequate? | Represen-tativeness of the Cases | Selection of Controls | Definition of Controls | Comparability of Cases and Controls on the Basis of the Design or Analysis | Ascertainment of exposure | Same method of ascertainment for cases and controls | Non-Response rate | Total scores |
| 1 | Stenzinger 2013 | ★ | ★ | **☆** | ★ | ★☆ | ★ | ★ | ★ | 7 |
| 2 | Li 2016 | ★ | ★ | **★** | ★ | ★★ | ★ | ★ | ☆ | 8 |
| 3 | Teramae 2014 | ★ | ★ | **☆** | ★ | ★☆ | ★ | ★ | ★ | 7 |
| 4 | Asaka 2015 | ★ | ★ | **☆** | ★ | ★★ | ★ | ★ | ★ | 8 |
| 5 | Jang 2009 | ★ | ★ | **☆** | ★ | ★☆ | ★ | ★ | ☆ | 6 |
| 6 | Shuang 2015 | ★ | ★ | **☆** | ★ | ★☆ | ★ | ★ | ☆ | 6 |
| 7 | Mvunta 2016 | ★ | ★ | **☆** | ★ | ★☆ | ★ | ★ | ★ | 7 |
| 8 | Zhang 2016 | ★ | ★ | **☆** | ★ | ★☆ | ★ | ★ | ★ | 7 |
| 9 | Feng 2016 | ★ | ★ | **★** | ★ | ★☆ | ★ | ★ | ★ | 8 |
| 10 | Kim 2013 | ★ | ★ | **★** | ★ | ★☆ | ★ | ★ | ★ | 8 |
| 11 | Noguchi 2013 | ★ | ★ | **★** | ★ | ★☆ | ★ | ★ | ★ | 8 |
| 12 | Yu 2013 | ★ | ★ | **☆** | ★ | ★☆ | ★ | ★ | ★ | 7 |
| 13 | Batra 2015 | ★ | ★ | **☆** | ★ | ★☆ | ★ | ★ | ★ | 7 |
| 14 | Chen 2014 | ★ | ★ | **☆** | ★ | ★☆ | ★ | ★ | ★ | 7 |
| 15 | He 2015 | ★ | ★ | **☆** | ★ | ★☆ | ★ | ★ | ★ | 7 |
| 16 | Feng 2017 | ★ | ★ | **★** | ★ | ★☆ | ★ | ★ | ★ | 8 |
| 17 | Noh 2013 | ★ | ★ | **☆** | ★ | ★☆ | ★ | ★ | ☆ | 6 |
| 18 | Jeh 2017 | ★ | ★ | **☆** | ★ | ★☆ | ★ | ★ | ★ | 7 |
| 19 | Jang 2008 | ★ | ★ | **★** | ★ | ★☆ | ★ | ★ | ★ | 8 |
| 20 | Ren 2016 | ★ | ★ | **★** | ★ | ★☆ | ★ | ★ | ★ | 8 |
| 21 | Nosho 2009 | ★ | ★ | **☆** | ★ | ★☆ | ★ | ★ | ☆ | 6 |
| 22 | Jang 2012 | ★ | ★ | **★** | ★ | ★☆ | ★ | ★ | ★ | 8 |
| 23 | Jung 2013 | ★ | ★ | **★** | ★ | ★☆ | ★ | ★ | ★ | 8 |
| 24 | Benard 2014 | ★ | ★ | **☆** | ★ | ★☆ | ★ | ★ | ★ | 7 |
| 25 | Chen 2014 | ★ | ★ | **★** | ★ | ★☆ | ★ | ★ | ★ | 8 |
| 26 | Lv 2014 | ★ | ★ | **☆** | ★ | ★☆ | ★ | ★ | ☆ | 6 |
| 27 | Lee 2015 | ★ | ★ | **☆** | ★ | ★☆ | ★ | ★ | ★ | 7 |
| 28 | Cheng 2016 | ★ | ★ | **★** | ★ | ★☆ | ★ | ★ | ★ | 8 |
| 29 | Chen 2011 | ★ | ★ | **☆** | ★ | ★☆ | ★ | ★ | ☆ | 6 |
| 30 | Jang 2012 | ★ | ★ | **★** | ★ | ★☆ | ★ | ★ | ★ | 8 |
| 31 | Hao 2014 | ★ | ★ | **☆** | ★ | ★☆ | ★ | ★ | ★ | 7 |
| 32 | Cheng 2016 | ★ | ★ | **☆** | ★ | ★★ | ★ | ★ | ★ | 8 |
| 33 | Li 2016 | ★ | ★ | **☆** | ★ | ★★ | ★ | ★ | ★ | 8 |
| 34 | Liu 2016 | ★ | ★ | **☆** | ★ | ★☆ | ★ | ★ | ★ | 7 |
| 35 | Cha 2009 | ★ | ★ | **☆** | ★ | ★☆ | ★ | ★ | ★ | 7 |
| 36 | Feng 2011 | ★ | ★ | **☆** | ★ | ★☆ | ★ | ★ | ☆ | 6 |
| 37 | Kang 2012 | ★ | ★ | **☆** | ★ | ★☆ | ★ | ★ | ★ | 7 |
| 38 | Noguchi 2014 | ★ | ★ | **☆** | ★ | ★☆ | ★ | ★ | ★ | 7 |
| 39 | Qiu 2016 | ★ | ★ | **☆** | ★ | ★★ | ★ | ★ | ★ | 8 |
| 40 | Szász 2016 | ★ | ★ | **☆** | ★ | ★★ | ★ | ★ | ★ | 8 |
| 41 | Zhang 2017 | ★ | ★ | **☆** | ★ | ★★ | ★ | ★ | ★ | 8 |
| 42 | Zhang 2013 | ★ | ★ | **☆** | ★ | ★☆ | ★ | ★ | ☆ | 6 |
| 43 | Ren 2017 | ★ | ★ | **☆** | ★ | ★★ | ★ | ★ | ★ | 8 |
| 44 | Shin 2016 | ★ | ★ | **☆** | ★ | ★☆ | ★ | ★ | ☆ | 6 |
| 45 | Zhang 2015 | ★ | ★ | **☆** | ★ | ★☆ | ★ | ★ | ★ | 7 |
| 46 | Cao 2014 | ★ | ★ | **☆** | ★ | ★★ | ★ | ★ | ★ | 8 |
| 47 | Jin 2015 | ★ | ★ | **☆** | ★ | ★☆ | ★ | ★ | ☆ | 6 |
| 48 | Kim 2015 | ★ | ★ | **☆** | ★ | ★☆ | ★ | ★ | ★ | 7 |
| 49 | Lee 2010 | ★ | ★ | **☆** | ★ | ★★ | ★ | ★ | ★ | 8 |
| 50 | Chung 2016 | ★ | ★ | **☆** | ★ | ☆☆ | ★ | ★ | ★ | 6 |
| 51 | Derr 2014 | ★ | ★ | **☆** | ★ | ★☆ | ★ | ★ | ☆ | 6 |
| 52 | Wu 2012 | ★ | ★ | **☆** | ★ | ★★ | ★ | ★ | ★ | 8 |
| 53 | Lee 2016 | ★ | ★ | **☆** | ★ | ★☆ | ★ | ★ | ☆ | 8 |
| 54 | Chung 2015 | ★ | ★ | **☆** | ★ | ★☆ | ★ | ★ | ☆ | 6 |
| 55 | Zhang 2016 | ★ | ★ | **☆** | ★ | ★☆ | ★ | ★ | ☆ | 6 |
| 56 | Sung 2010 | ★ | ★ | **☆** | ★ | ★☆ | ★ | ★ | ☆ | 6 |
| 57 | Gharabaghi 2016 | ★ | ★ | **☆** | ★ | ★☆ | ★ | ★ | ☆ | 6 |
| 58 | Li 2015 | ★ | ★ | **☆** | ★ | ★☆ | ★ | ★ | ★ | 7 |
| 59 | Lin 2015 | ★ | ★ | **☆** | ★ | ★★ | ★ | ★ | ★ | 8 |
| 60 | Noh 2013 | ★ | ★ | **☆** | ★ | ★☆ | ★ | ★ | ★ | 7 |
| 61 | Zhang 2013 | ★ | ★ | **☆** | ★ | ☆☆ | ★ | ★ | ★ | 6 |
| 62 | Chen 2014 | ★ | ★ | **☆** | ★ | ★★ | ★ | ★ | ★ | 8 |
| 63 | Grbesa 2015 | ★ | ★ | **☆** | ★ | ☆☆ | ★ | ★ | ★ | 6 |

^a^ A maximum of 2 stars can be allotted in this category, one for age, the other for other controlled factors.
