# Supplementary figures and images for: Survival and Clinicopathological Significance of SIRT1 Expression in Cancers: A Meta-Analysis
Source: Front Endocrinol (Lausanne). 2019 Mar 13;10:121. doi: 10.3389/fendo.2019.00121 (PMC6424908; doi:10.3389/fendo.2019.00121)

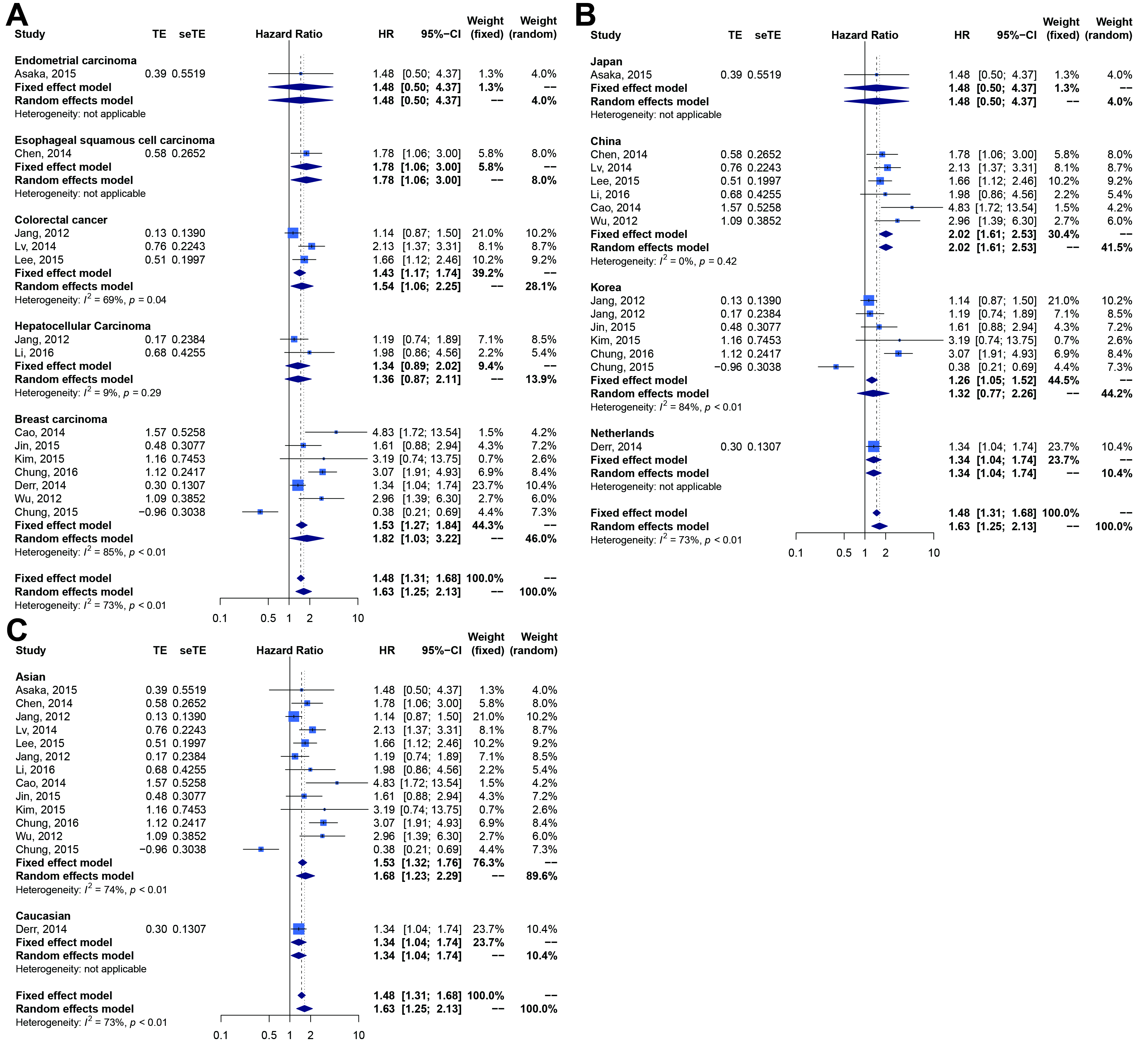

Supplement: Supplementary Figure 2 — Forest plot of subgroup analysis for SIRT1 overexpression and DFS in cancers. (A) Cancer subgroup, (B) Country subgroup, (C) Ethnicity subgroup. [file Image_2.TIF]

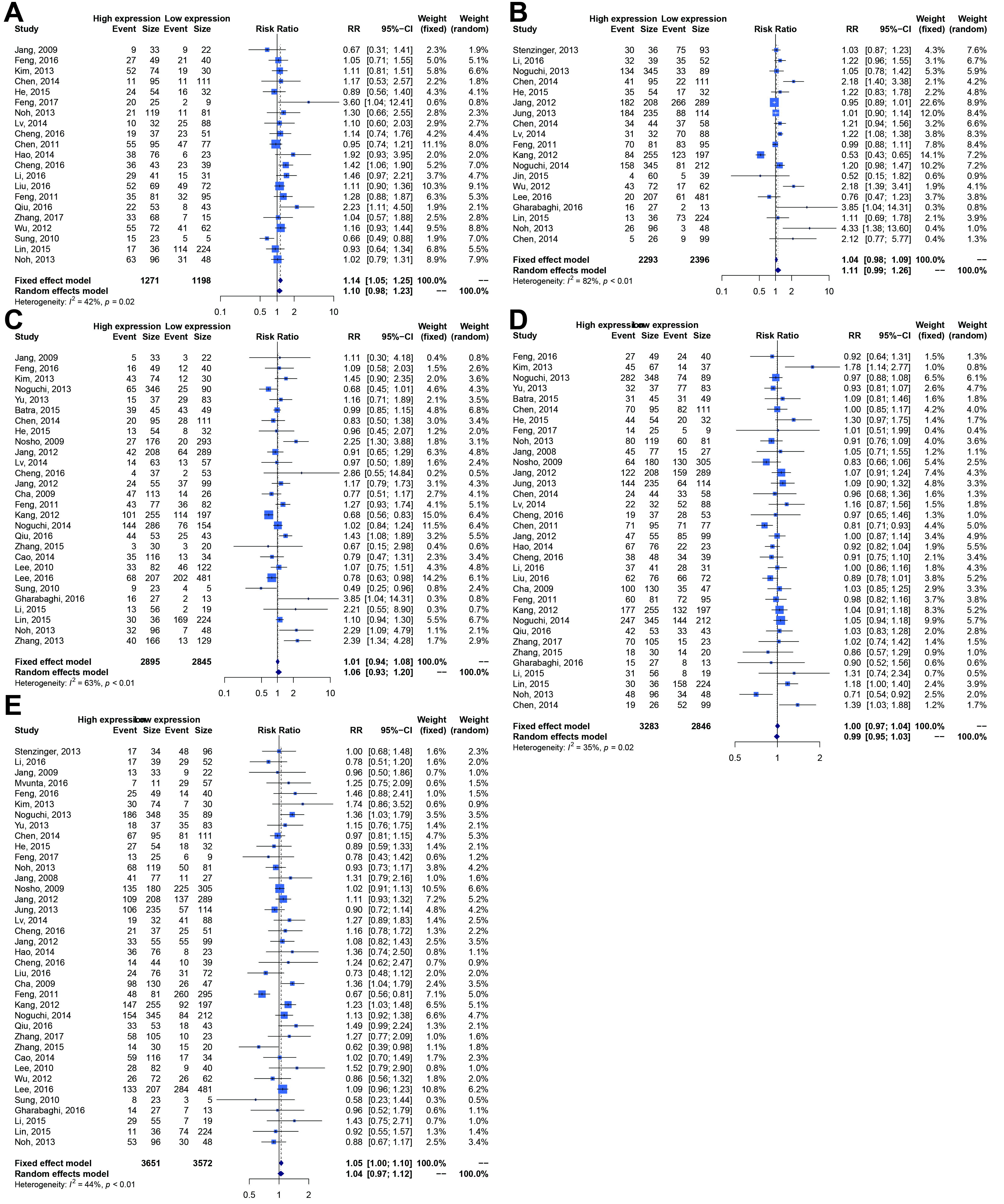

Supplement: Supplementary Figure 3 — Forest plots of non-essential clinicopathological outcomes compared SIRT1 overexpression with underexpression. (A) Tumor size, (B) Depth of tumor invasion, (C) Differentiation, (D) Gender, (E) Age. [file Image_3.TIF]

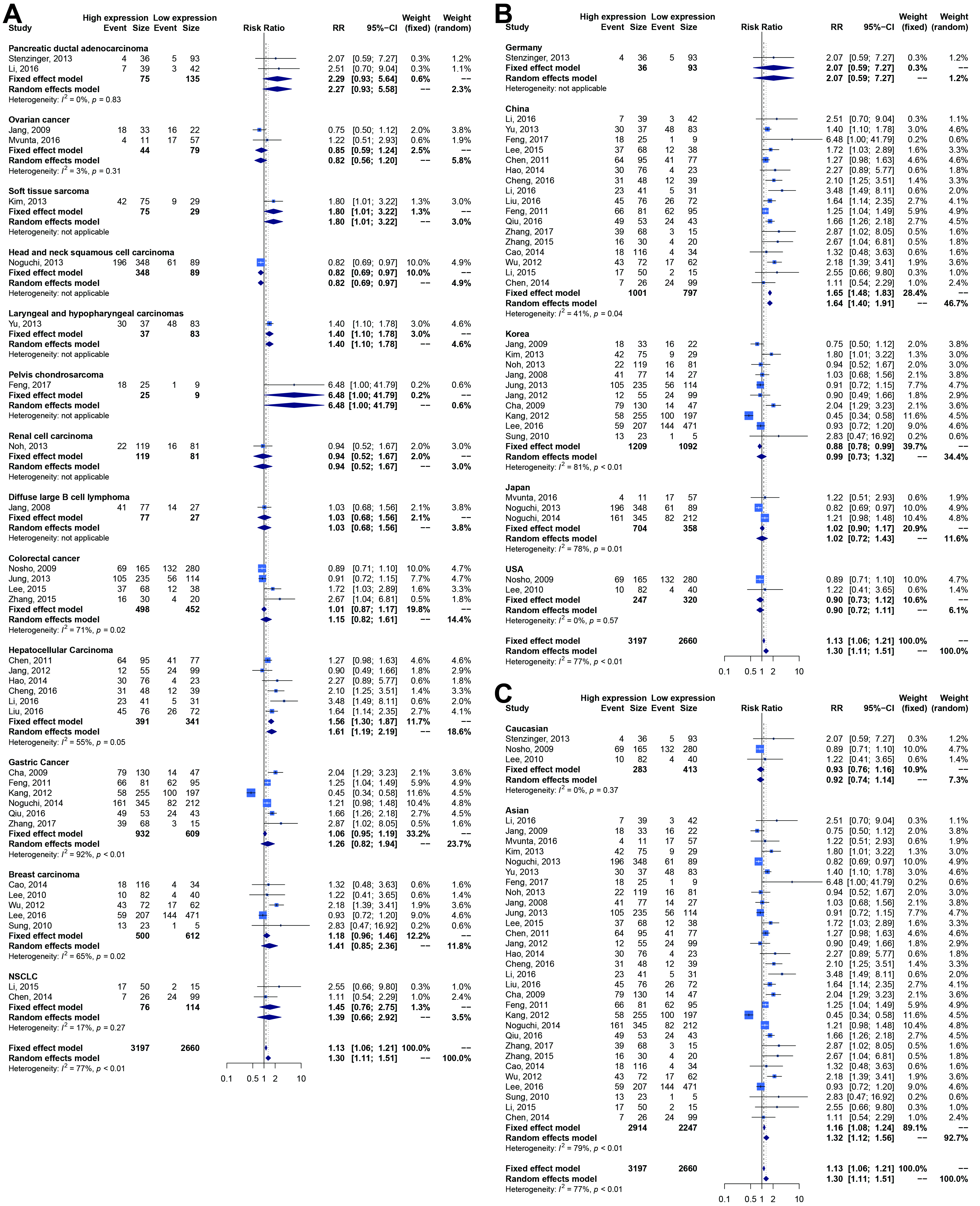

Supplement: Supplementary Figure 4 — Forest plot of subgroup analysis for SIRT1 overexpression and TNM stage in cancers. (A) Cancer subgroup, (B) Country subgroup, (C) Ethnicity subgroup. [file Image_4.TIF]

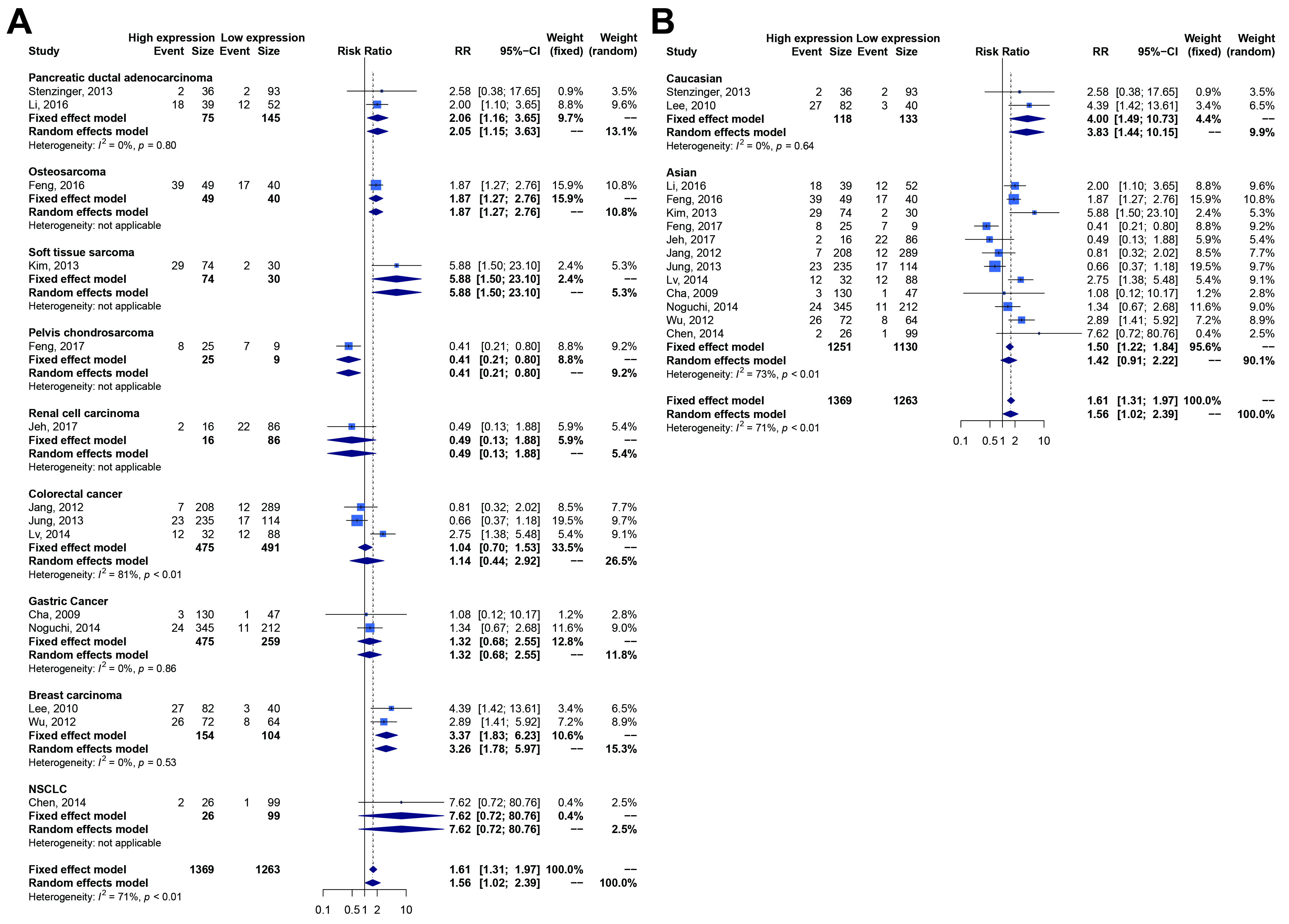

Supplement: Supplementary Figure 5 — Forest plot of subgroup analysis for SIRT1 overexpression and distant metastasis in cancers. (A) Cancer subgroup, (B) Ethnicity subgroup. [file Image_5.TIF]

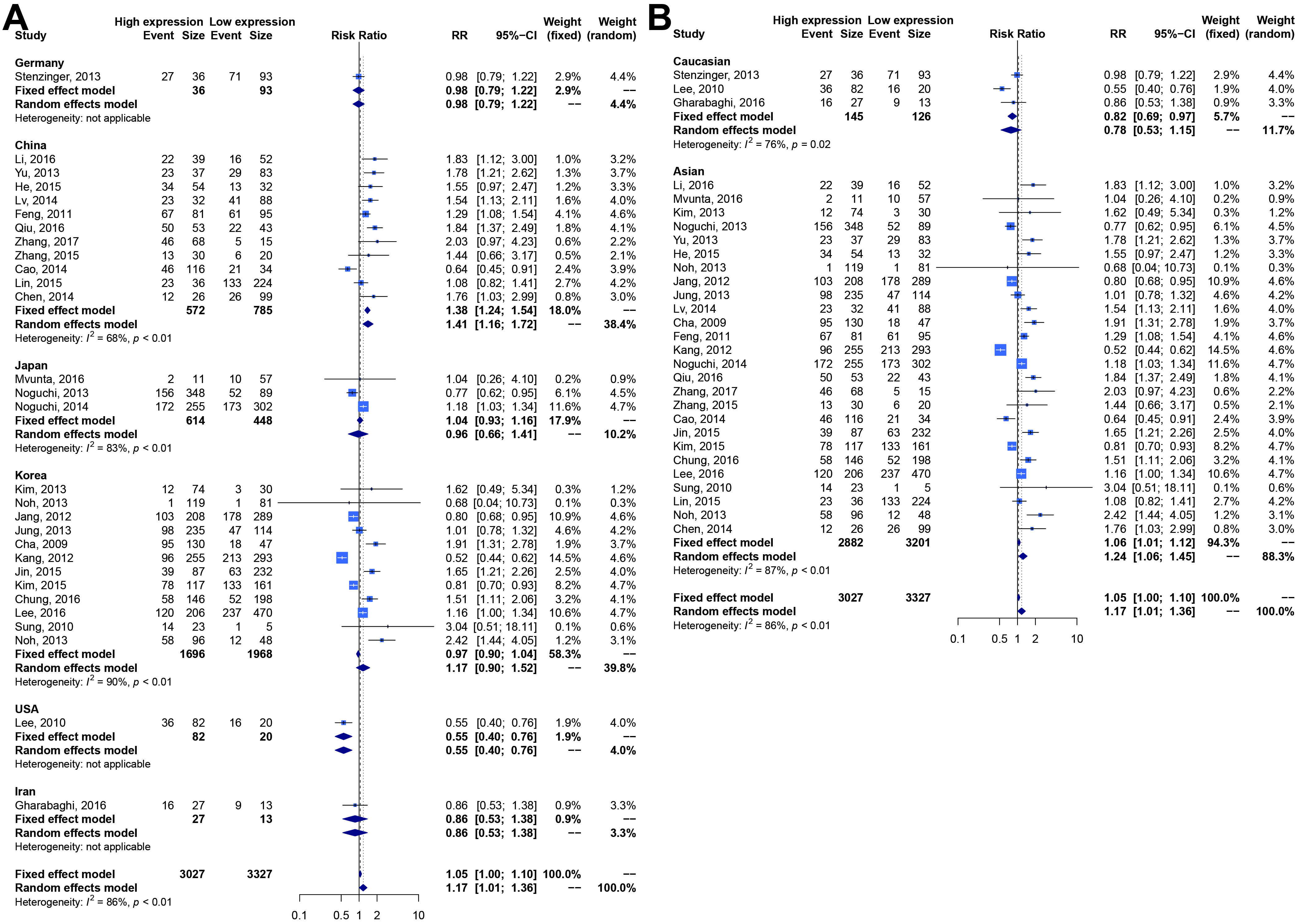

Supplement: Supplementary Figure 6 — Forest plot of subgroup analysis for SIRT1 overexpression and lymphatic metastasis in cancers. (A) Country subgroup, (B) Ethnicity subgroup. [file Image_6.TIF]

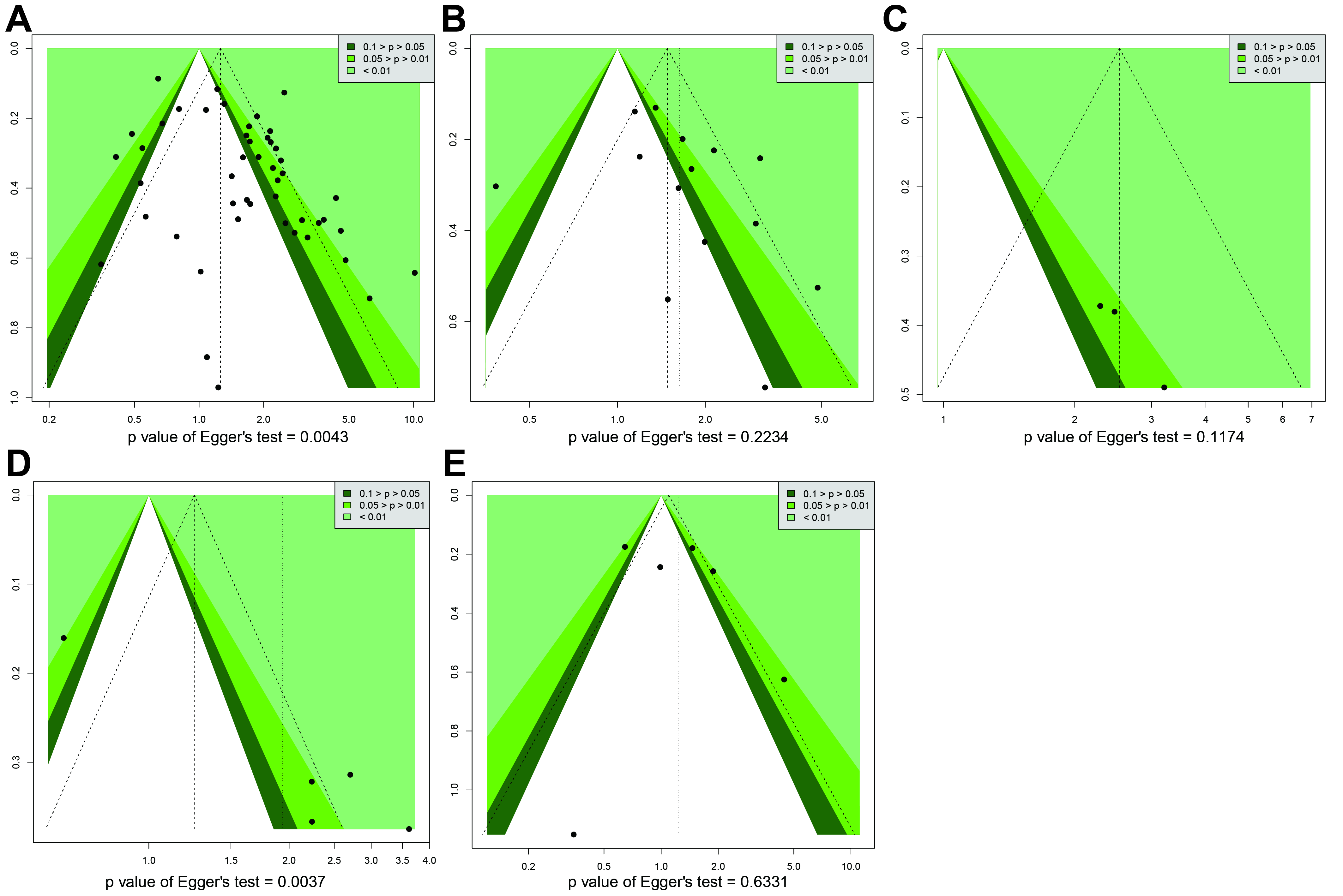

Supplement: Supplementary Figure 7 — Funnel plot for publication bias for SIRT1 expression and prognosis. (A) OS, (B) DFS, (C) EFS, (D) RFS, (E) CCS. [file Image_7.TIF]

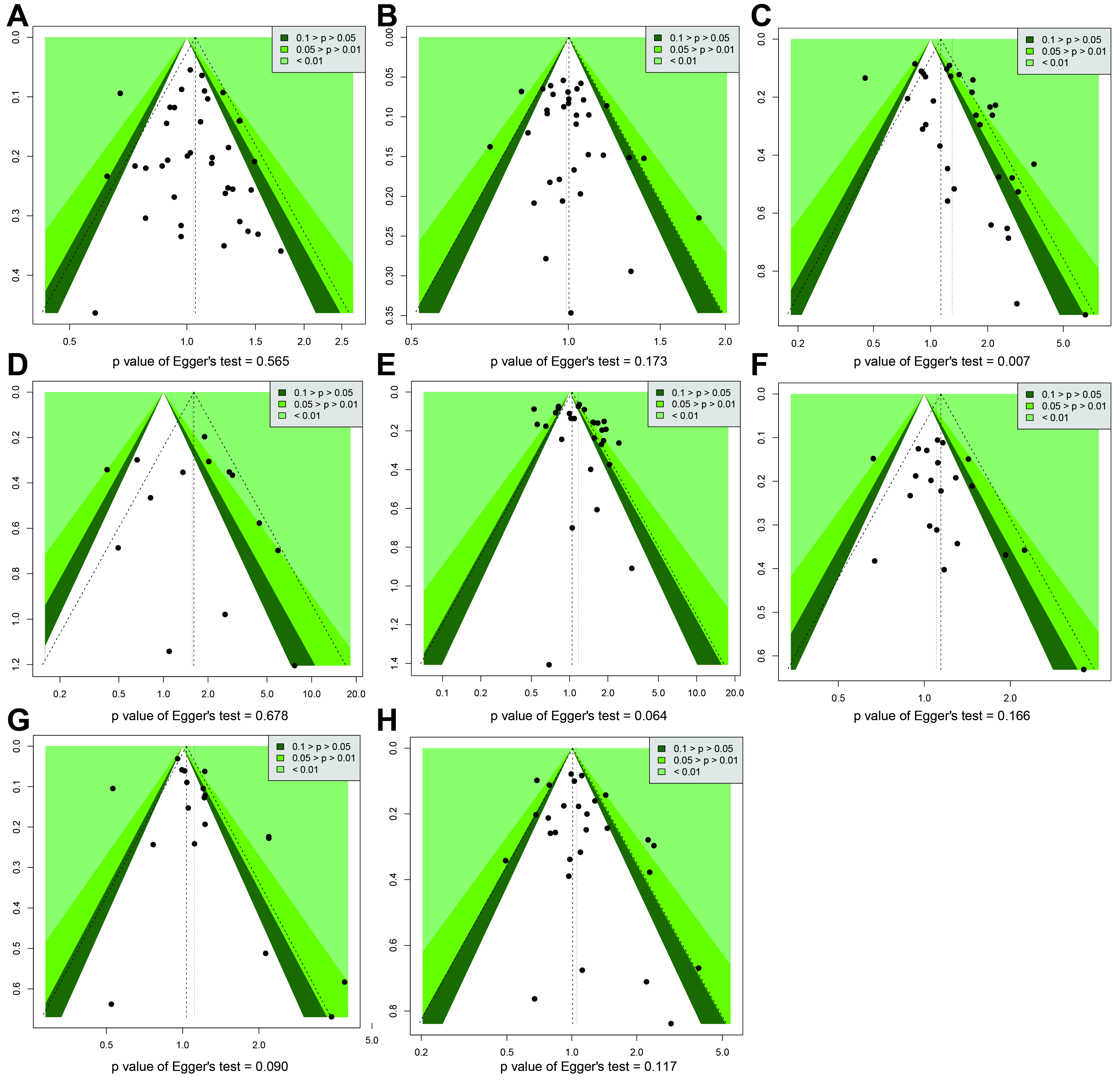

Supplement: Supplementary Figure 8 — Funnel plot for publication bias for SIRT1 expression and clinicopathological characteristics. (A) Age, (B) Gender, (C) Tumor stage, (D) Distant metastasis, (E) Lymphatic invasion, (F) Tumor size, (G) Depth of tumor invasion, (H) Differentiation. [file Image_8.TIF]
